# Supplementary material for: Phasor approach of Mueller matrix optical scanning microscopy for biological tissue imaging
Source: Biophys J. 2021 Jul 3;120(15):3112–25. doi: 10.1016/j.bpj.2021.06.008 (PMC8390965; doi:10.1016/j.bpj.2021.06.008)
Supplement: Document S1. Supporting materials and methods, Figs. S1–S3, and Tables S1 and S2 [file mmc1.pdf]

Biophysical Journal, Volume *120*

Supplemental information

Phasor approach of Mueller matrix optical scanning microscopy for biological tissue imaging

Aymeric Le Gratiet, Luca Lanzano, Artemi Bendandi, Riccardo Marongiu, Paolo Bianchini, Colin Sheppard, and Alberto Diaspro

Appendix

Aymeric Le Gratiet[1,*], Luca Lanzano[2], Artemi Bendandi[1,3,4], Riccardo Marongiu[1,3], Paolo Bianchini[1], Colin J.R. Sheppard[1,5], Alberto Diaspro[1,3]

7th June 2021

[1] Nanoscopy and NIC@IIT, Istituto Italiano di Tecnologia, Via Enrico Melen 83, 16152 Genova, Italy
[2] Department of Physics and Astronomy "Ettore Majorana", University of Catania, Via S. Sofia, 64, 95123 Catania, Italy
[3] DIFILAB, Department of Physics, University of Genoa, Via Dodecaneso 33, 16146 Genova, Italy
[4] CONCEPT, Istituto Italiano di Tecnologia, Via Enrico Melen 83, 16152 Genova, Italy
[5] School of Chemistry, University of Wollongong, Wollongong NSW 2522, Australia
* Corresponding author: aymeric.legratiet@iit.it

Averaging the three linear polarization state inputs

The three linear polarization state inputs are oriented at 0° (H), 90° (V) and 45° and generate the phasor plots presented Fig.2. In order to simplify the interpretation of the linear and RCP phasors for the general case, we averaged the phasor plots of the linear polarization states related to the H phasor. The relationship between H and V corresponds to a simple phase shift equal to π with the same modulation, resulting in the new (g,s) coordinates for V

$$g = M * cos(\phi^V + \pi), \tag{1}$$

$$s = M * sin(\phi^V + \pi). \tag{2}$$

To shift the phasor from the 45° to the H input, we have to consider a phase shift and a translation through the s axis in function of R, giving the new coordinates for 45° as

$$g = M * cos(\phi^{45} - \pi/2), \tag{3}$$

$$s = M * sin(\phi^{45} - \pi/2) + 0.5 * (1 + cos(R)). \tag{4}$$

Applying these mathematical transformations, the V and 45° present the same phasor with H, allowing the visualization of the average phasor spots.

Expressions of (R, α_R) in function of (M, ϕ)

Tab.S1 resumes the polarization transformation after interaction with the retarder for R = π, R = $\pi/2$ and R = $\pi/4$ for two orientations of the optical fast axis, such as α_R = 0° and α_R = 45°. It can be predicted by the Stokes-Mueller formalism [1], given by the product of the birefringent sample Eq.28 with the input Stokes vector Eq.12 and Eq.15.

Retarder value \ Input polarization		H(1 1 0 0)	CR(1 0 0 1)
**R = π**	α_R= 0°	(1 1 0 0)	(1 0 0 -1)
	α_R= 45°	(1 -1 0 0)	(1 0 0 -1)
**R = $\pi/2$**	α_R= 0°	(1 1 0 0)	(1 0 1 0)
	α_R= 45°	(1 0 0 1)	(1 -1 0 0)
**R = $\pi/4$**	α_R= 0°	(1 1 0 0)	(1 0 1/√2 -1/√2)
	α_R= 45°	(1 1/√2 0 -1/√2)	(1 -1/√2 0 -1/√2)

Table 1: Output Stokes vectors from the simulated retarder of the three retardance cases R = π, $\pi/2$, $\pi/4$ for $\theta = 0°$ and 45° at each input polarization states. The blue shapes correspond to the polarization ellipse.

On one hand, by comparing Tab.1 and Fig.3, the (g,s) coordinates are related to the output polarization states after interaction with the sample by

$$(g, s) = (M * cos(\phi), M * sin(\phi)) = 0.5 * (cos(2\chi).cos(2\psi), cos(2\chi).sin(2\psi)), \quad (5)$$

where χ is the ellipticity of the polarization ellipse ($-\pi/4 \leq \chi \leq \pi/4$), obtained from M = 0.5*cos(2χ) and $\phi = 2\psi$. From an ellipsometric point of view [2], these expressions of g and s are functions of the output Stokes coefficients as

$$(g, s) = 0.5 * (S_1, S_2). \quad (6)$$

Thus, the absolute value of S_3 is retrieved by the expression

$$S_3 = \sqrt{1 - S_1^2 - S_2^2} = sin(2\chi). \quad (7)$$

By converting the MM into the phasor, we lost the direction of rotation of the polarization states defined by the sign of S_3. However, it is always possible to image the S_3 coefficient at the same time as the ones from the modulation amplitude and phase. Thus, the expression of ψ and χ are

$$\psi = 0.5 * tan^{-1}\left(\frac{s}{g}\right), \quad (8)$$

$$\chi = 0.5 * tan^{-1}\left(\frac{S_3}{\sqrt{s^2+g^2}}\right). \tag{9}$$

It is worth noting that the drawback of using the (ψ,χ) images instead of the RCP phasor is the need of using S_3.
On the other hand, from the Mueller matrix formalism, the expression of the transformed polarized light after interaction with the sample can be obtained as a Stokes vector (S_0,S_1,S_2,S_3) using the set of Eqs.12-15 and the MM of the retarder Eq.28. Thus, we can express the Stokes coefficients S_1 and S_2 in function of R and α_R for the RCP input

$$S_1 = sin(R).sin(2\alpha_R), \tag{10}$$

$$S_2 = -sin(R).cos(2\alpha_R). \tag{11}$$

In this way, the last set of equations for the RCP input gives a direct link between (g,s) coordinates and (R, α_R) as follows

$$(g,s) = 0.5*(S_1,S_2) = 0.5*(sin(R).sin(2\alpha_R), sin(R).cos(2\alpha_R)). \tag{12}$$

By identifying this last equation with the modulation and phase from the (g,s) coordinates, we show that by simply reading the coordinates (g,s) in the RCP phasor plot, we have the following relation:

$$M = 0.5*sin(R). \tag{13}$$

It is worth noting that for the phase, the relation is

$$cos(\phi) = sin(\alpha_R) = cos(2\alpha_R + \pi/2), \tag{14}$$

thus,

$$\phi = 2\alpha_R + \pi/2. \tag{15}$$

Pure dichoic medium

The phasor plots for a pure dichroic sample are presented Fig.S1 for the linear and RCP polarization inputs.

For $\alpha_D = 0°$, the modulation value for the H input is smaller than pure retarder cases and becomes maximum (g,s) = (0,0.5) for $\alpha_D < 45°$. In parallel for the RCP input, the modulation is maximum for any orientation but translates from s = 0 to g = 0 in the positive phasor coordinates region, contrary to a pure retarder case (from g = 0 to s = 0 in the negative phasor coordinates region). These two patterns could be used for discriminating between pure retarder and dichroic media. Additionally, the relation between ϕ and α_D is simply

$$\phi = 2 \times \alpha_D \tag{16}$$

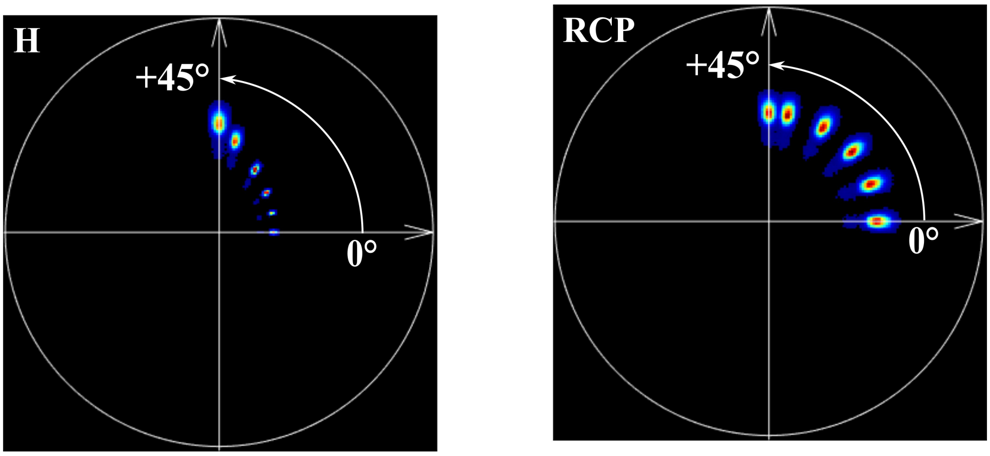

Figure S 1. Phasor plots for a pure dichroic medium at the H and RCP polarization inputs.

Experimental MM				Lu-Chipman
1.000	−0.027	0.006	0.003	$D = 0.028$
0.008	0.204	0.059	−0.011	$R = 14.85°$
0.084	0.014	0.639	0.010	$\alpha_R = 44.43°$
−0.002	0.058	0.017	0.065	$P_d = 0.396$
1.000	−0.043	0.002	0.001	$D = 0.043$
0.036	0.294	0.089	0.059	$R = 6.34°$
−0.038	0.089	0.758	0.004	$\alpha_R = -38.89°$
−0.003	0.021	−0.024	0.039	$P_d = 0.479$

Table 2: Simulated MM of a mixed medium composed by (top) dichroic oriented at 30° and a retarder $\pi/4$ oriented at 30° and (bottom) a retarder $\pi/4$ oriented at 30° and dichroic oriented at 30°.

However, a mixed medium composed by a pure dichroic and a pure retarder leads to a complicated case where the associated polarimetric parameters could be only retrieved by performing several measurements at different sample orientation.

Mixed dichroic and retarder medium

LC decomposition is based on modeling the propagation of the light through a successive arrangement of elementary optical features composed successively by a dichroic, a birefringent and a depolarizer. However, modifying the order with this method leads to misinterpret the real polarimetric signature of the sample and results different value of the parameters. As a demonstration, we evaluate the MM and the associated LC decomposition of a medium composed by a dichroic oriented at 30° followed by a retarder $\pi/4$ oriented at 30°. Then, we extract the MM and the associated polarimetric parameters when this order is inverted as presented Tab.2.

In the meantime, the phasor plot of the two type of media are identical as presented in

Fig.S2.

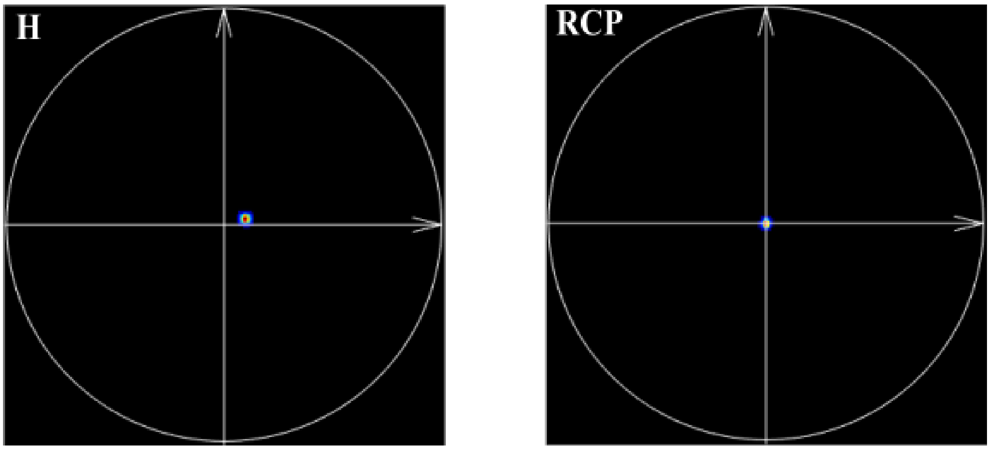

Figure S 2. H and RCP Phasor plots for a mixed medium composed by dichroic oriented at 30° and a retarder $\pi/4$ oriented at 30°.

In summary, we demonstrated that the ordering of the discrete optical features that are used in LC to model the sample is crucial for interpreting the fingerprint of the sample, while there is no influence on the phasor plots since this approach doesn't require any a prio knowledge of the sample.

Pure depolarizing medium

For a depolarizing MM, we calculated the resulting phasor from the R and RCP phasor plot at different value of P_d lying from 1.0 (no depolarizing) down to 0.2 (highly depolarizing) in Fig. 3. We presented the phasor in the case of a mixed polarimetric medium composed by a 90° retarder oriented at 0° and a depolarizer defined by a depolarization index $P_d = 1$, 0.85, 0.65 and 0.27. As a comparison, the phasor plot in the case of a pure retarder is shown Fig.S3(a) and corresponds to the first phasor spot in Fig.S3(b). The case of a pure depolarizer is presented Fig.S3(c) for $P_d = 1$, 0.75, 0.5 and 0.2.

Here, we show that the presence of depolarization affects the position of the phasor spot, that results a decreasing of the modulation of the when P_d drops down. In the case of a mixed polarimetric signature birefringent-depolarizer Fig.S3(b), the decreasing of the modulation reduces the contrast between the different phases that could affect the SNR for determining the retardance and its orientation, limiting such approach for highly scattering media. However, it is worth noting that the combined reading of both the H and RCP phasors allows the discrimination overall the possible mixture of retardance and depolarization. For instance, MM-phasor is still capable of distinguishing between a specimen having a small retardance with no depolarization and another one having a high retardance and a strong depolarization. In the extreme case $P_d = 0$ Fig.S3(c), the H and RCP phasor gives both a spot located at the origin, which differs from air sample that is seen at (g,s) = (0.5,0) in the H phasor. A remarkable property of the MM-phasor in dealing with depolarization media is that the (g,s) coordinates for a specific depolarizer is similar of multiplying it by the depolarization factor. Indeed, the Fig.S3(c) gives g = 0.5, 0.38, 0.25 and 0.10 which corresponds to multiplying gmax = 0.5 by Pd = 1.0, 0.75, 0.5 and 0.2, respectively. This simplifies the analysis of the retardance and its

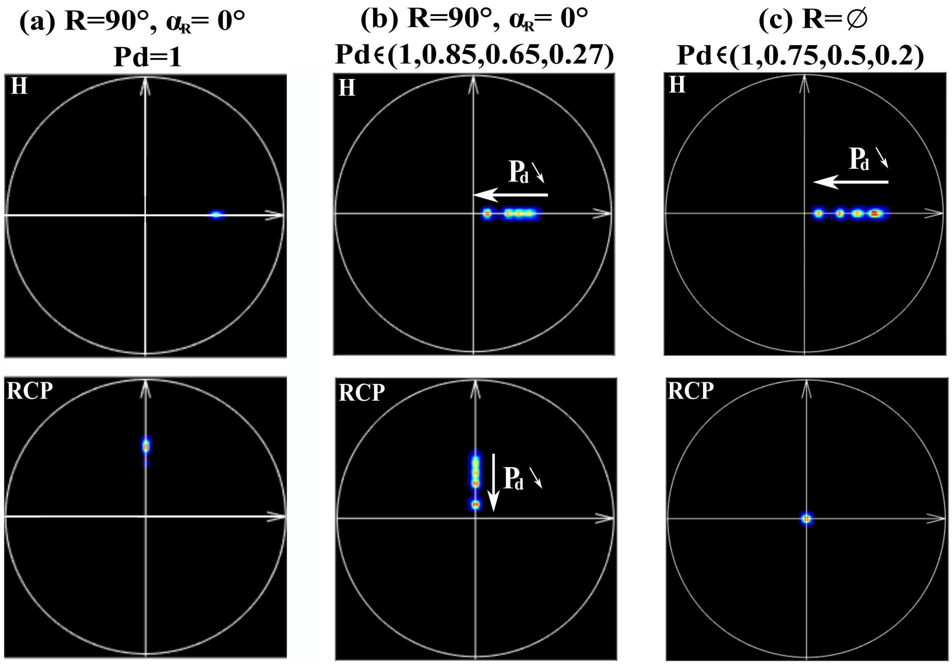

Figure S 3. Phasor plots for (a) A pure retarder (R = 90°, α_R = 90°), (b) a mixed retarder (R = 90°, α_R = 90°) and depolarizer medium and (c) a pure depolarizer. The values for the depolarization index P_d are 1, 0.85,0.65 and 0.27 for (b) and 1, 0.75, 0.5 and 0.2 for (c).

orientation from the phasor plot in the case of depolarization, which is typically the case for biomedical diagnosis of pathological tissue.

References

[1] S. Cloude, “Group theory and polarization algebra,” *Optik (Stuttg)*, vol. 75, pp. 26–36, 1986.

[2] R. Azzam, “The intertwined history of polarimetry and ellipsometry,” *Thin Solid Films*, vol. 519, no. 9, pp. 2584–2588, 2011.
