# Supplementary material for: Phasor approach of Mueller matrix optical scanning microscopy for biological tissue imaging
Source: Biophys J. 2021 Jul 3;120(15):3112–25. doi: 10.1016/j.bpj.2021.06.008 (PMC8390965; doi:10.1016/j.bpj.2021.06.008)
Supplement: Document S2. Article plus supporting material [file mmc2.pdf]

Biophysical Journal
Article

Biophysical Society

Phasor approach of Mueller matrix optical scanning microscopy for biological tissue imaging

Aymeric Le Gratiet,[1,*] Luca Lanzano,[1,2] Artemi Bendandi,[1,3,4] Riccardo Marongiu,[1,3] Paolo Bianchini,[1] Colin Sheppard,[1,5] and Alberto Diaspro[1,3]

[1]Nanoscopy and NIC@IIT, Istituto Italiano di Tecnologia, Genova, Italy; [2]Department of Physics and Astronomy "Ettore Majorana", University of Catania, Catania, Italy; [3]DIFILAB, Department of Physics, University of Genoa, Genova, Italy; [4]CONCEPT Lab, Istituto Italiano di Tecnologia, Genova, Italy; and [5]School of Chemistry, University of Wollongong, Wollongong, Australia

ABSTRACT Mueller matrix microscopy is an advanced imaging technique providing a full characterization of the optical polarization fingerprint of a sample. The Lu-Chipman (LC) decomposition, a method based on the modeling of elementary polarimetric arrangements and matrix inversions, is the gold standard to extract each polarimetric component separately. However, this models the optical system as a small number of discrete optical elements and requires a priori knowledge of the order in which these elements occur. In stratified media or when the ordering is not known, the interpretation of the LC decomposition becomes difficult. In this work, we propose a new, to our knowledge, representation dedicated to the study of biological tissues that combines Mueller matrix microscopy with a phasor approach. We demonstrate that this method provides an easier and direct interpretation of the retardance images in any birefringent material without the use of mathematical assumptions regarding the structure of the sample and yields comparable contrast to the LC decomposition. By validating this approach through numerical simulations, we demonstrate that it is able to give access to localized structural information, resulting in a simple determination of the birefringent parameters at the microscopic level. We apply our novel, to our knowledge, method to typical biological tissues that are of interest in the field of biomedical diagnosis.

SIGNIFICANCE Mueller matrix microscopy provides a full characterization of the optical polarization fingerprint of a sample without using any dyes. The gold standard method for extracting the elementary optical components is based on the Lu-Chipman decomposition and has proven its efficiency in quantifying the main polarimetric parameters as the dichroism, birefringence, and scattering. In this work, we demonstrate that combining Mueller matrix microscopy with a phasor approach provides a direct interpretation of the retardance images in any birefringent material without modeling. We believe that this work is a starting point for the interpretation of the localized polarimetric fingerprint inside tissues in a direct approach, leading to future applications for in situ diagnosis of pathologies and diseases that could assist histopathological evaluation.

INTRODUCTION

Mueller matrix (MM) microscopy is a label-free method based on the control of generated and analyzed polarization states, providing a full characterization of the optical polarization fingerprint of a sample (1). At least 16 intensities are needed to completely determine the 4×4 matrix of the sample, in which each independent element m_{ij} can be associated with a specific physical phenomenon, such as linear or circular dichroism, birefringence, and scattering (2–5).

Submitted October 2, 2020, and accepted for publication June 4, 2021.

*Correspondence: aymeric.legratiet@iit.it

Editor: Anne Kenworthy.

https://doi.org/10.1016/j.bpj.2021.06.008

© 2021 Biophysical Society.

This is an open access article under the CC BY license (http://creativecommons.org/licenses/by/4.0/).

MM microscopy has been successfully used in numerous research fields such as materials science (6), biomedical diagnostics (7–9), and microscopic imaging (10–13), showcasing its versatility and easy implementation on multimodal systems (14). To highlight a particular physical process, decomposing the MM is a mandatory step for tracking the amplitude of a specific polarimetric effect. Among all the decomposition methods, the gold standard Lu-Chipman (LC) polar decomposition is based on a priori knowledge of the sample and models the propagation of the light through a successive arrangement of elementary optical features composed of a dichroic, a birefringent element, and a depolarizer. This method, based on the Stokes-Mueller formalism, uses multiple matrix inversions that drastically

increase the estimated systematic errors, so the physical meaning of the MM could be misinterpreted. Additionally, aside from this decomposition, numerous works propose manual modification of the order of the elementary optical components to match the most realistic conditions (15,16). This step requires a priori knowledge of the sample and can be arduous, as finding the proper decomposition corresponding to the physical polarimetric fingerprint in the case of complex and mixed biological media can be particularly challenging. Based on these issues, modifying the order of the optical features that model the sample properties results in wrong polarimetric values, disconnected from the real composition of the sample.

The phasor plot approach has been extensively applied to graphically analyze spectra in a variety of advanced microscopy techniques. It was originally introduced in the framework of fluorescence lifetime imaging (FLIM) analysis, as an alternative to multiexponential decay fitting (17). In the phasor FLIM approach, the fluorescence decay at each pixel is analyzed in the frequency domain via a Fourier transform algorithm that generates, for each pixel (x, y) and for a given frequency, a complex number (g,s) (17). The values of (g, s) associated with all the pixels of an image are usually represented in a two-dimensional histogram, called the phasor plot. This graphical representation of FLIM data has gained increasing popularity among FLIM users, as it allows identifying multiple molecular species (18) and quantifying Förster resonance energy transfer interactions (19) without any prior fluorescence decay model assumptions (20,21). More recently, the phasor plot approach has been extended to the analysis of other types of microscopy data, including spectral images (22), image correlation spectroscopy data (23,24), and super-resolution images (25,26). In the context of nonlinear, label-free microscopy, the phasor plot has been used to analyze polarization-resolved second harmonic generation data and map the features of collagen architecture in tissues (27). Moreover, a recent theoretical work has proven the phasor space based on the analysis of circular polarized light scattering to be an intuitive tool, sensitive to the chiral organization of biopolymers at the submicroscopic level (28).

Here, we combine MM microscopy with the phasor approach to facilitate the interpretation of MM imaging of biological samples. In our method, named MM-phasor, the full MM is first converted into four Stokes vector images, and then a Fourier transform algorithm is applied to each of these images. In this way, the coupling of both techniques allows an alternative interpretation of the multiparametric polarimetric contrast at a molecular level, independent of the sample position, giving similar values to the LC decomposition. Moreover, we demonstrate numerically that MM-phasor is able to retrieve the retardance value in the case of randomly layered, oriented birefringent materials. This new, to our knowledge, approach is a model-free method for accessing some of the polarimetric information, whereas LC fails because of the requirement for a priori ordering of the successive optical features of the sample for such MM decomposition. For this purpose, we discuss the sensitivity of such an approach for discriminating between structures of interest that exhibit similar contrast. As a proof of concept, we apply this approach to imaging of biological specimens (collagen and myosin fibers) showing mostly birefringence. Such materials exhibit negligible dichroism and/or depolarization at the microscopic level because of their high transparency and weak scattering. For this reason, in this work we briefly consider the influence of these two effects on the phasor map. Finally, to understand the influence of the polarized light on sample orientation, we propose decoupling the effect of both the input and output polarized light from the sample optical orientation.

MATERIALS AND METHODS

MM optical scanning microscope

The experimental setup was a modified inverted commercial laser scanning microscope Olympus FV1000 (Tokyo, Japan), reported in our previous work (13). The excitation source is a laser diode at 808 nm. Polarization information is encoded by four polarization states (0, 90, and 45° and right circular polarization) from a motorized polarization states generator (PSG) placed before the microscope body, composed of a linear polarizer, a half-wave plate, and a quarter-wave plate. To image the samples in a reflection configuration, we placed a silver plane reflective mirror at the focal plane of a 10×/0.4 NA objective. The passive polarization states analyzer (PSA) analyzed the transformation of the polarization after the interaction of the polarized light with the sample. It consisted of a thin glass slice, a Fresnel rhomb, two Wollaston prisms, and four photodiodes to collect the signal pixel by pixel for four polarization states. The four input polarization states generation and the four photodiodes were synchronized with the scanning unit via a custom LabVIEW routine via a data board acquisition. Finally, a MATLAB (The MathWorks, Natick, MA) program was used to extract the full MM image of the sample from the 16 acquired polarization-resolved 256 × 256 images.

As we have shown in our previous work (13), the calibration procedure includes taking into account the remaining optical orientation misalignment of the PSG and PSA, as well as the polarimetric signature of the optical devices in the microscope body, composed mainly of lenses and reflective mirrors. It is based on the eigenvalue calibration method to calibrate the PSG and PSA using reference elements such as air, linear polarizer, and quarter-wave plate (29). The accuracy of acquiring the full MM is determined by the condition number of the polarimeter (PSG and PSA without microscope); in this study, this corresponds to condition number = 2.6, presenting uncertainty less than 5% on the Mueller coefficients. Next, we image the MM of the whole system to take into account the polarimetric fingerprint of the microscope pixel by pixel over the entire field of view. Thereby, MM images of the sample are obtained by directly inverting the microscope fingerprint by applying

$$\left[M_{sample}(x,y)\right] \approx \left[M_{mes}(x,y)\right] \times \left[M_{\mu}(x,y)\right]^{-1}, \qquad (1)$$

where $[M_{sample}(x, y)]$, $[M_{mes}(x, y)]$, and $[M_{\mu}(x, y)]$ are the MM images of the sample, of the total system microscope + sample, and of the microscope only, respectively. This equation neglects the influence of optical components that follow the sample in the reflection path, as discussed in our previous work (13). Furthermore, we have demonstrated that the double pass of light through the sample in the epigeometry imaging causes a π-dephasing effect at the reflection on the mirror surface. Thus, the retardance value

$R_{measured}$ from the double-pass optical path is defined as 2π, so that the exact value R_{exact} is recovered by applying $R_{exact} = 2\pi - R_{measured}$. Considering that the samples are nondepolarizing, the increase of the scattering through the sample after the double pass is considered in the standard deviation (SD).

MM image acquisition and analysis

The physical effects are extracted from an implemented LC decomposition algorithm (1). It is based on the representation of the sample by the three main optical interactions successively crossed by the excitation light: dichroism, birefringence, and scattering. These interactions correspond to the product of the three matrices associated with the polarimetric parameters:

$$[M_{sample}] \approx [M_\Delta] \times [M_R] \times [M_D], \tag{2}$$

where $[M_\Delta]$, $[M_R]$, and $[M_D]$ are the sample matrices describing the total depolarization, diattenuation (linear and circular), and birefringence (linear and circular), respectively. After normalization by the total transmitted light, corresponding to the m_{00} element, the physical parameters denoted in this work are retrieved by

$$P_d = \sqrt{\frac{\sum_{i,j=0}^{3} m_{ij}^2 - m_{00}^2}{3m_{00}^2}}, \tag{3}$$

$$R = \arccos[\mathrm{tr}(M_R)/2 - 1], \tag{4}$$

$$\alpha_R = 0.5 \times \arctan(r_2/r_1), \tag{5}$$

and

$$D = \sqrt{m_{01}^2 + m_{02}^2 + m_{03}^2}, \tag{6}$$

where P_d, R, and D correspond to the depolarization index (30), retardance, and diattenuation; m_{ij} are the MM elements; $\mathrm{tr}(M_R)$ is the trace of the retardance matrix M_R; and (r_1, r_2) are the elements of the retardance vector (31). In this way, each of these three physical parameters varies between $0 \le P_d \le 1$, $0° \le R \le 180°$, and $0 \le D \le 1$ and is used for coding the post-treated images pixel by pixel. It is worth noting that the amplitudes of these three parameters are directly related to the sample properties. The values of orientation corresponding to a weak signal of diattenuation and retardance are not physical and are coded in black in the α_R images.

The MM and the associated LC decomposition data are presented in Table 1, averaged over the region of interest (ROI) 1 and ROI 2 for air and a transparent adhesive film area, respectively, from Fig. 6.

As shown in Table 1, the calculated values match with the expected values for air and transparent film. Indeed, the expected values for a nondepolarizing medium ($P_d = 1$) such as air are $D = 0$, $R = 0°$, and $P_d = 1$, whereas for the transparent film they are $D = 0$, $0° \le R \le 180°$, and $P_d = 1$. It is worth noting that the MM of transparent film presents higher MM coefficient values related to the dichroism induced by the interface of the layer. The wider SD for the transparent film, especially for α_R, comes from the inhomogeneity of the surface for such a sample.

The phasor plot analysis described in Result and discussion: MM-phasor analysis method section was implemented in MATLAB (The MathWorks). To remove residual biological components and clean the phasor plots from background contributions, a ROI was defined selecting only the fingerprint of the fiber.

Sample preparation

Muscular tissue was harvested from the leg of a raw rabbit purchased in a supermarket and was cleaned from other connective tissue. The muscular tissue was rinsed several times with phosphate-buffered saline solution (pH 7.4) and fixed with 4% paraformaldehyde in phosphate-buffered saline solution overnight. The day after, the tissue was dehydrated by using a series of ethanol solutions of increasing alcoholic concentration. Next, the sample was embedded in paraffin before sectioning using a microtome (Leica, Wetzlar, Germany). The thickness of the muscle sections was of 5 μm. The sections were placed and fixed on a microscope slide and imaged.

RESULTS AND DISCUSSION

MM-phasor analysis method

In our previous work, we developed a full MM optical scanning microscope based on the sequential generation of four polarization states 0, 90, and 45° and right circular polarization states (RCPs) through the PSG (13). The polarized radiation can be described by a 1×4 Stokes vector $\vec{S} = (S_0, S_1, S_2, S_3)$, where the coefficients S_0, S_1, S_2, and S_3 are obtained from the combination of polarized light intensities $I_{0°}$ (horizontal), $I_{90°}$ (vertical), $I_{45°}$, $I_{135°}$, I_{RCP} (right circular), and I_{LCP} (left circular), giving the following set of equations (32):

TABLE 1 Experimental normalized MM of homogeneous air and transparent adhesive film samples and associated LC polarimetric parameters averaged over the ROIs

Experimental MM	LC
$\begin{bmatrix} 1.000 & 0.003\pm0.009 & -0.009\pm0.011 & 0.012\pm0.022 \\ -0.005\pm0.024 & 0.999\pm0.022 & -0.002\pm0.025 & -0.008\pm0.049 \\ 0.011\pm0.019 & 0.007\pm0.018 & 1.002\pm0.025 & -0.011\pm0.042 \\ 0.007\pm0.014 & -0.009\pm0.010 & 0.004\pm0.012 & 1.014\pm0.031 \end{bmatrix}$	$D = 0.027 \pm 0.014$<br>$R = 1.9 \pm 0.9°$<br>$P_d = 1.007 \pm 0.01$
$\begin{bmatrix} 1.000 & 0.012\pm0.009 & -0.005\pm0.011 & 0.071\pm0.029 \\ -0.012\pm0.029 & 1.023\pm0.032 & -0.222\pm0.026 & -0.327\pm0.063 \\ 0.007\pm0.054 & 0.376\pm0.024 & 0.678\pm0.026 & 1.022\pm0.078 \\ -0.023\pm0.016 & 0.055\pm0.019 & -0.342\pm0.018 & 0.562\pm0.036 \end{bmatrix}$	$D = 0.072 \pm 0.026$<br>$R = 55.5 \pm 1.9°$<br>$\alpha_R = -85.7 \pm 0.9°$<br>$P_d = 1.051 \pm 0.029$

MM values taken from Fig. 6, ROIs 1 and 2.

$$S_0 = I_{0^\circ} + I_{90^\circ} = I_{45^\circ} + I_{135^\circ} = I_{LCP} + I_{RCP}, \quad (7)$$

$$S_1 = I_{0^\circ} - I_{90^\circ}, \quad (8)$$

$$S_2 = I_{45^\circ} - I_{135^\circ}, \quad (9)$$

and

$$S_3 = I_{RCP} + I_{LCP}. \quad (10)$$

The transformation of each input polarization state after interaction with the sample is recorded pixel by pixel by the PSA allowing the determination of the 16-element MM of the sample. The link between the input and output polarization states, $\vec{S_{in}}$ and $\vec{S_{out}}$ respectively, is established using the MM of the sample $[M_{sample}(x, y)]$:

$$\vec{S_{out}} = \left[M_{sample}\right] \times \vec{S_{in}}. \quad (11)$$

In the case in which an optical block is added between the PSG and PSA, the polarimetric signature of this feature must be taken into account. More particularly, the output Stokes vector can be directly retrieved as a function of input experimental polarization states $[W] = (\vec{w_H}, \vec{w_V}, \vec{w_{45^\circ}}, \vec{w_{RCP}})$ by

$$\vec{S_{out}}^{H} = \left[M_{sample}(x,y)\right].\vec{w_H}, \quad (12)$$

$$\vec{S_{out}}^{V} = \left[M_{sample}(x,y)\right].\vec{w_V}, \quad (13)$$

$$\vec{S_{out}}^{45^\circ} = \left[M_{sample}(x,y)\right].\vec{w_{45^\circ}}, \quad (14)$$

and

$$\vec{S_{out}}^{CR} = \left[M_{sample}(x,y)\right].\vec{w_{RCP}}, \quad (15)$$

where $(\vec{w_H}, \vec{w_V}, \vec{w_{45^\circ}}, \vec{w_{RCP}})$ are the experimental input Stokes vectors linear horizontal, linear vertical, linear 45°, and RCP polarization states.

First, to convert the MM into a phasor plot, we reconstruct the linear polarization-resolved intensity from the output Stokes vectors of Eqs. 12, 13, 14, and 15. Indeed, the linear output polarization states intensities I_θ ($\theta = 0$, 45, 90, or 135°) can be extracted directly from each previous Stokes vector defined by Eqs. 7, 8, 9, and 10 by

$$I_{0^\circ}(x,y) = 0.5 \times [S_0(x,y) + S_1(x,y)], \quad (16)$$

$$I_{90^\circ}(x,y) = 0.5 \times [S_0(x,y) - S_1(x,y)], \quad (17)$$

$$I_{45^\circ}(x,y) = 0.5 \times [S_0(x,y) + S_2(x,y)], \quad (18)$$

and

$$I_{135^\circ}(x,y) = 0.5 \times [S_0(x,y) - S_2(x,y)]. \quad (19)$$

Analyzing the polarization-resolved signal I_θ is similar to rotating the polarization analyzer after the sample, with the aim of following the anisotropic polarization emission of molecules under illumination, as widely realized for studying nonlinear anisotropic molecular orientation (33). Because we follow the linear polarization dependence, the circular polarization intensity is omitted but can be extracted from the combination of the $S_0(x, y)$ and $S_3(x, y)$ Stokes coefficients.

The phasor P(x, y) associated with the polarization-resolved image $I_\theta(x, y)$ has coordinates $g(x, y)$ and $s(x, y)$ given by

$$g(x,y) = \frac{\sum I_\theta \cos(2\pi\theta/\Theta)}{\sum I_\theta(x,y)}, \quad (20)$$

and

$$s(x,y) = \frac{\sum I_\theta \sin(2\pi\theta/\Theta)}{\sum I_\theta(x,y)}, \quad (21)$$

where $\Theta = 180°$ and $1/\Theta$ is the frequency used for the Fourier transform. Thus, every single pixel can be identified as a vector in phasor space with two phasor coordinates (g, s) mapped to (x, y) coordinates. The step-by-step protocol for converting the MM signal into a phasor, for each pixel, is summarized in Fig. 1. In general, from the phasor approach, it is possible to analyze multiple frequencies in the signal. In this work, we have limited our analysis to a single frequency. The phasor is a vector of modulus M and angle ϕ given by

$$M = \sqrt{s^2 + g^2}, \quad (22)$$

and

$$\phi = \arctan\left(\frac{s}{g}\right). \quad (23)$$

The parameters M and ϕ are usually called modulation and phase and, as we shall see, are important in the interpretation of the output polarimetric signal.

The phasor coordinates (g, s) can be calculated back from M and ϕ from

$$g = M \times \cos(\phi), \quad (24)$$

and

$$s = M \times \sin(\phi). \quad (25)$$

In the field of FLIM, the phasor approach defined M to be between 0 and 1 and ϕ between 0 and π (17). Here, from the Stokes-Mueller formalism, the orientation of the optical fast

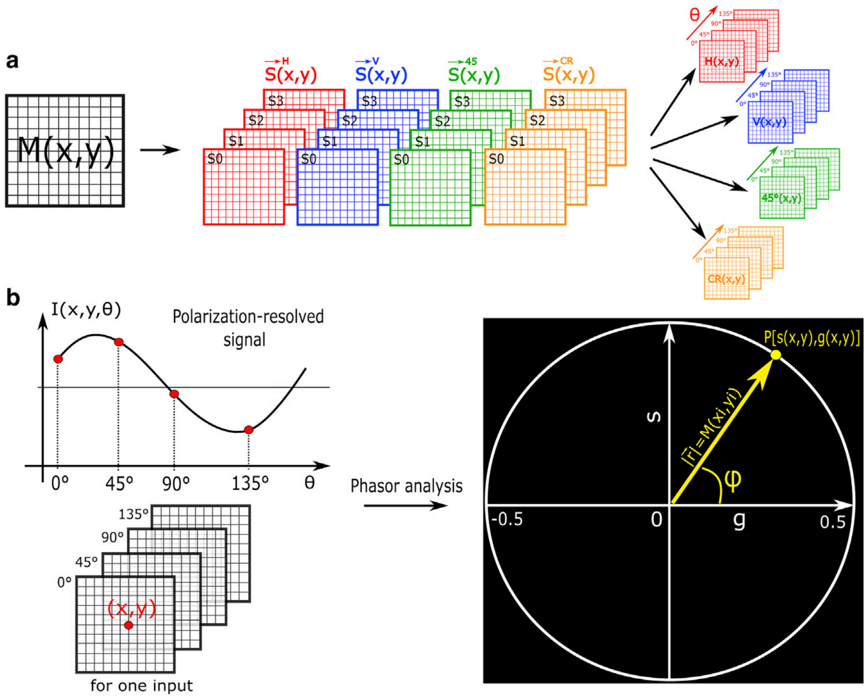

FIGURE 1 Schematic diagram of the protocol for converting the MM image into a phasor plot. (*a*) The MM image conversion into Stokes-Mueller images, then giving linear polarization images for input polarization states H, V, 45°, and RCP. (*b*) For a given input polarization state, a stack of images of the modulated linear polarized light signal is built pixel by pixel (x, y), which can be plotted in the phasor space at the coordinates (g, s).

axis of the sample is defined between $-\pi$ and π, modifying the definition range of $M \in [0; 0.5]$ and $\phi \in [-\pi; \pi]$ (1,34). Finally, the (g, s) coordinates are calculated pixel by pixel from the polarization-resolved images using Eqs. 20 and 21. In this work, the two-dimensional histogram of the values $g(x, y)$ and $s(x, y)$ associated with each pixel are then represented in the phasor plot, in which the phasor spot is coded from red to blue, for the highest to the lowest bin. A pixel-by-pixel phasor representation could also be used, but then imaging contrast information is lost, and it becomes arduous distinguishing the location of each component of the image.

Numerical simulation on birefringent materials

To study the phasor plot changes as a function of the polarization input, we simulate the behavior in phasor space of well-known reference samples that are easy to assemble experimentally, for instance, air and birefringent materials.

Air sample

First, we simulate the phasor signature from the air sample, i.e., without placing a specimen in the point spread function (PSF). To have comparable results with experimental data, we add the statistical SD measured experimentally and reported in Table 1 in Materials and methods. Thus, the corresponding air MM is given by

$$M_{air}(x, y) = I_4 + \Delta(x, y), \tag{26}$$

where I_4 is the 4×4 identity matrix and $\Delta(x, y)$ is the additional error matrix corresponding to the experimental SD of the microscope. By applying Eqs. 20 and 21, the phasor plots for the air sample for each input polarization states are presented in Fig. 2.

The simulated air sample exhibits the properties of the input excitation light because of the absence of any interaction with polarimetric objects. The H and V polarization state inputs reveal a phasor in the positive ($\phi = 0°$) and negative ($\phi = \pi$) x axis, respectively. Considering that the electric field oscillates in the perpendicular orientation between these two polarization states, this means that the phasor defined the polarization orthogonality at π. This is confirmed by the 45° input, which exhibits a phasor at $\phi = \pi/2$ from the H polarization states input in the positive y axis. This behavior depends on the period used for the Fourier transform ($\Theta = 180°$). Indeed, the observed polarization angle ψ (i.e., the angle at which the polarimetric signal has a peak) is the angle of the output polarized light propagation plane with the (x, y) laboratory axis, equal to $\psi = 0°$, 45°, and 90° for the H, 45°, and V output polarization states, respectively. It is related to the phase ϕ in the phasor space by the relationship

$$\psi = \phi(\theta / 2\pi) = \phi/2. \tag{27}$$

Thus, the phase of the MM-phasor has a range $-\pi \leq \phi \leq \pi$, corresponding to the range $-\pi/2 \leq \psi \leq \pi/2$ for the linear polarization angle. Here, the modulus in the absence of any anisotropic materials is equal to $M = 0.5$ for all inputs. In addition, the RCP input shows a phasor centered at the origin, indicating that the polarized light interacts homogeneously and has no preferential orientation, as predicted.

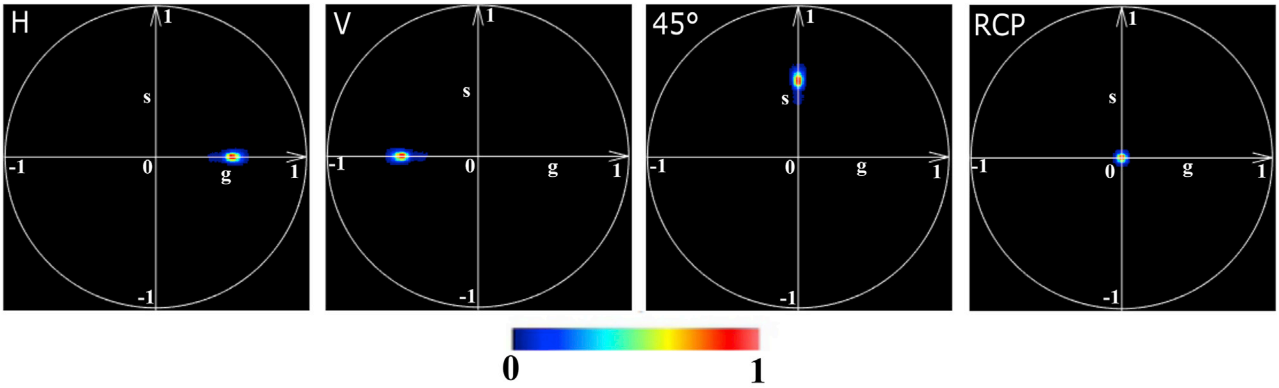

FIGURE 2 Phasor plot of a simulated air sample for each of the four polarization state inputs: H (linear horizontal), V (linear vertical), 45° (linear 45°), and RCP (right circular polarization).

Birefringent sample

In this work, we study only the MM-phasor of a simulated birefringent material. Indeed, birefringence is the main polarimetric parameter, presenting the highest imaging contrast in biological tissues. We simulated the phasor plot of a birefringent material of retardance R and optical fast axis orientation α_R, using the following MM:

$$M_{retarder} = \begin{bmatrix} 1 & 0 & 0 & 0 \\ 0 & \cos^2(2\alpha_R) + \sin^2(2\alpha_R) \times \cos(R) & \cos(2\alpha_R) \times \sin(2\alpha_R) \times (1-\cos(R)) & \sin(2\alpha_R) \times \sin(R) \\ 0 & \cos(2\alpha_R) \times \sin(2\alpha_R) \times (1-\cos(R)) & \sin^2(2\alpha_R) + \cos^2(2\alpha_R) \times \cos(R) & -\cos(2\alpha_R) \times \sin(R) \\ 0 & -\sin(2\alpha_R) \times \sin(R) & \cos(2\alpha_R) \times \sin(R) & \cos(R) \end{bmatrix}, \quad (28)$$

with

$$R = \frac{2\pi \times \Delta n \times e}{\lambda}, \quad (29)$$

where $\Delta n = (n_e - n_o)$ is the birefringence, e is the thickness of the retarder, and λ is the excitation wavelength.

The phasor plots are presented in Fig. 3 for different R-values as a function of α_R for the linear and circular input polarization states. To improve the visibility of each spot on the phasor plot, we kept the same signal/noise ratio (SNR) as for air, and the orientation angle corresponds to 0, 10, 20, 30, 40, and 45°. To simplify the phasor representation, we show only the H and RCP phasor plots. We discuss in the Supporting materials and methods how to merge these into a single phasor plot by averaging the three linear polarization inputs (H, V, and 45°).

First, we consider the H phasor plot. For $\alpha_R = 0°$, the phasor is identical to that obtained without a sample in Fig. 2. The reason for this is that there is no polarization change when the input polarized light is aligned with the fast axis of the birefringent object, in accordance with the polarization formalism (1). For $R = \pi$, the phasor rotates toward π as the value of α_R increases from 0 to 45° (Fig. 3 *a*). For $R < \pi$, from $R = 3\pi/4$ to $R = \pi/4$ (Fig. 3, *b–d*), the phasor follows a similar rotation in function of α_R, but the center of this rotation changes as a function of R (Fig. 3, *b–d*, *top*). This center translates until the limit case $R = 0$, in which the phasor becomes identical to the air phasor. Thus, in the H phasor plot, there is no straightforward relation between the values of R and α_R and the values of M and ϕ. If we consider the RCP phasor plot, for $R = \pi$, the RCP phasor is identical to the air phasor, whatever the value of α_R. For $R < \pi$, the phasor rotates from the angle $\phi = \pi/2$

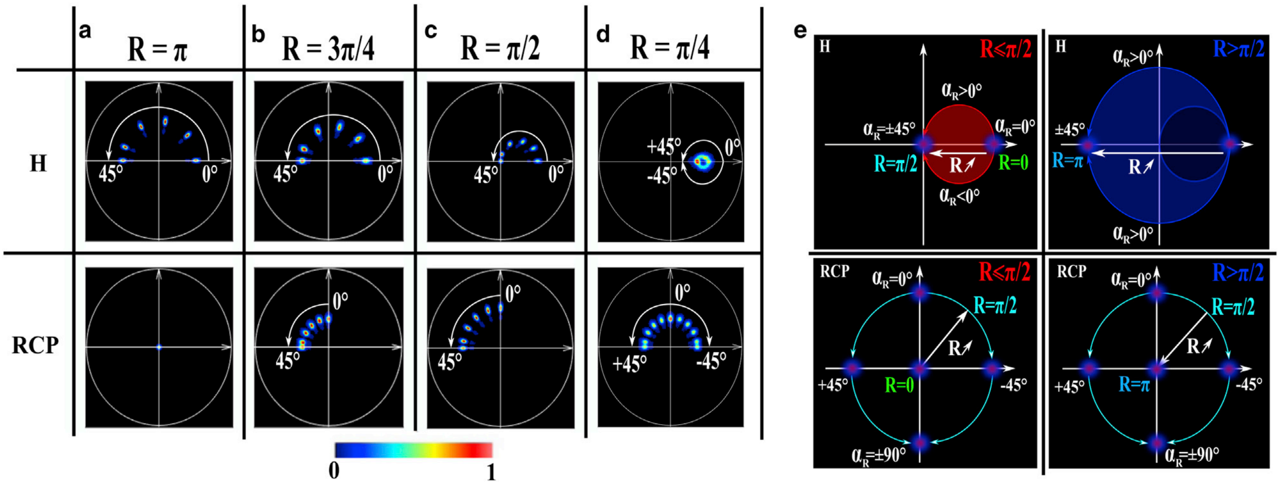

FIGURE 3 Phasor plots of a simulated retarder for linear and circular polarization input states as a function of the optical fast axis orientation α_R (between 0 and 45° in steps of 10°) for a retardance of (*a*) π, (*b*) $3\pi/4$, (*c*) $\pi/2$, and (*d*) $\pi/4$. (*e*) Schematic diagram of the phasor plot pattern for a retarder as function of the R and α_R parameters for linear and circular polarization input states.

($\alpha_R = 0°$) to the angle $\phi = \pi$ ($\alpha_R = 45°$) for any value of R (Fig. 3, *b–d*, bottom). Thus, for $R < \pi$, the retarder orientation α_R is linked directly to the phase angle ϕ as (see Supporting materials and methods)

$$\alpha_R = \frac{\phi}{2} - \frac{\pi}{4}, \tag{30}$$

where the phasor angle ϕ is defined between $-\pi/2$ and $3\pi/2$, we get values of α_R between $-\pi/2$ and $\pi/2$. The modulation observed at the RCP phasor is independent of the optical fast axis orientation of the retarder and depends only on R (Fig. 3, *b–d*, *bottom*). Thus, for $R < \pi$, the retardance R is linked directly to the phasor modulation M by

$$R = \arcsin(2 \times M), \tag{31}$$

where the modulation M varies between 0 and 0.5, corresponding to R-values defined between 0 and $\pi/2$. For any given value of modulation M, another valid solution is

$$R' = \pi - \arcsin(2 \times M). \tag{32}$$

In other words, in the RCP phasor we get the same modulation M for the retardance values R and $\pi - R$ (see, for instance, Fig. 3, b and *d*). Notably, we can discriminate between the cases $R < \pi/2$ and $R > \pi/2$ by additionally reading the H phasor. For $R < \pi/2$, the phasor lies within the small circle of center (0.25, 0) and radius 0.25 (area inside the *red line* in Fig. 3 *e*, *top left*), whereas for $R > \pi/2$, the phasor lies outside the small circle and within the circle of center (0, 0) and radius 0.5 (area inside the *blue line* in Fig. 3 *e*, *top right*).

In summary, as presented in Fig. 3 *e*, we numerically demonstrated the capability of the MM-phasor to extract R and α_R from the RCP and H phasor plots for any type of birefringent specimen. For $0 < R < \pi$, these polarimetric parameters are obtained by simply extracting M and ϕ in both H and RCP phasors based on Eqs. 30, 31, and 32). For the specific case $R = \pi$, only the H phasor is needed for obtaining α_R, as $\alpha_R = \phi/4$ (Fig. 3 *e*, *top*), which speeds up the analysis. However, the limitation of this approach comes when determining the values from a weak birefringent material, resulting in M-values too small compared to the SNR. The reason is that the phasor spot evolves too close around the center and is assimilated to the case in which the sample is only air. We calculated the modulation value as a function of R for determining the sensitivity of MM-phasor in Fig. 4 at the same orientation $\alpha_R = 0°$. This is similar to finding the minimal retardance in which the phasor spot is included in the experimental SD reported for the air sample. As explained in the previous section, the phasor spot lies on the g axis with $s = 0$. The simulated data have been extracted from the RCP phasor because R is extracted from this space as demonstrated in Eq. 32. Hence, we have presented the modulation value for a retardance from $\pi/2$ to $\pi/40$, resulting in M-values between 0.5 (maximal M) and 0.03 as previously discussed above.

In the Materials and methods, the measured SD leads to an uncertainty on the air M-value equal to 0.05. This means that below this modulation, the phasor spot provided by a weak birefringent specimen can be assimilated to the air, therefore comprising the resolution limit of the MM-phasor approach. From Fig. 4, it can be seen that $M = 0.05$ is reached for $R = \pi/31 \sim 5.8°$. Thus, the MM-phasor method is not able to discriminate structures of interest that exhibit a contrast below this limit, comparable to the SNR reported in Table 1 for the LC decomposition.

Layered birefringent fibers

To characterize the performance of the MM-phasor approach in the case of a histopathological sample, we simulate a structure composed of a successive arrangement of elementary randomly oriented birefringent features. The

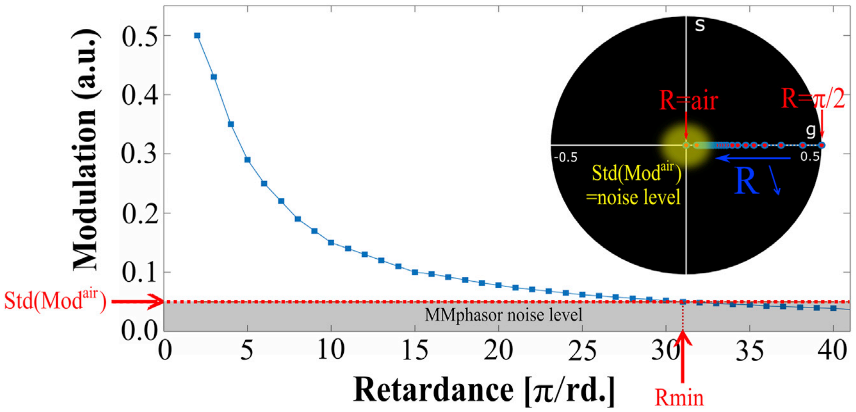

FIGURE 4 Evolution of the modulation value as a function of the inverse retardance as a multiple of π and the corresponding raw simulated data. The data are calculated in RCP phasor space, with retardance between $\pi/2$ (= 90°) and $\pi/40$ (= 4.5°), with the same orientation $\alpha_R = 0°$. a.u., arbitrary unit.

model shown in Fig. 5 *a* is basically related to the situation one encounters in the presence of a pathology that has induced a disorganization of the connective tissue. The modeling of such a medium consists of simply multiplying the retarder MMs reported in Eq. 28 with the same *R*-value but at different orientations. Here, we simulate an image containing one, two, three, or four sets of simulated fibers, calculate (g, s) pixel by pixel from this image and the associated phasor plot, and then extract the retardance from this plot. In our model, shown in Fig. 5 *a*, the medium under illumination corresponds to a successive arrangement of birefringent components with the same thickness *e* and oriented at 0, 10, 20, and 30°. To make a comparison between the LC and the phasor approach in realistic conditions, we consider a type I collagen fiber with a thickness of $e = 50$ μm and $\Delta n = 0.0017$ (35). Thus, *R* is calculated from Eq. 29 and provides the ground truth of the simulation, equal to $R_1 = 37°$. We calculate the MMs for a single zero-degree layer and then add the differently oriented layers one by one. The determination of *R* between the two approaches is compared to the ground truth as a function of the number of fibers in the illumination volume and is presented in Fig. 5 *b*. Noting that the value of R providing in this section corresponds to a single point in the birefringent fiber with a specific thickness and without dimension in the *xy* plane (perpendicular to the PSF volume).

In the case of a single fiber oriented at 0°, both LC and MM-phasor approaches provide a good agreement with the expected value $R_1 = 37°$. By adding a second layer and a third one, oriented at 10 and 20°, the expected values are $2 \times R_1 = 74°$ and $3 \times R_1 = 111°$. Thus, we notice a strong dispersion by using the LC approach, which yields 93 and 141°, whereas the MM-phasor approach is still consistent with the expected values, 68 and 121°. By adding a fourth fiber oriented at 30°, the expected value is $4 \times R_1 = 148°$ and the MM-phasor approach is closer to the real value of 149°, whereas the LC

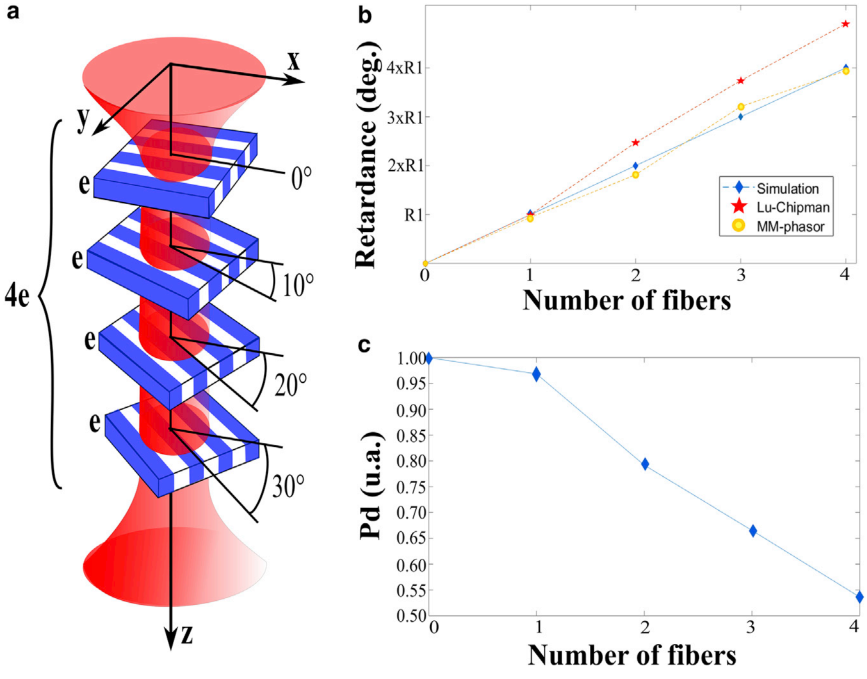

FIGURE 5 (*a*) Schematic of the layered oriented birefringent medium. Each layer has a thickness of $e = 50$ μm and is oriented at 10° compared to the previous one. (*b*) Retardance value, as a multiple of $R_1 = 37°$, as a function of the number of fibers in the illumination volume. The blue, red, and orange plots correspond to *R*-values calculated from the ground truth, the LC decomposition, and the MM-phasor approach, respectively. (*c*) Depolarization index, P_d, calculated from the LC decomposition, as a function of the number of fibers in the illumination volume.

fails by giving 185°. The reason for the nonconsistency of the LC decomposition when the complexity of the sample increases is that the model composed of an arrangement of a dichroic, birefringent element and a depolarizer is no longer suitable. As shown above, the ordering of the optical components is crucial for physically interpreting the MM of the sample, and inverting it drastically influences the value of the polarimetric parameters, as illustrated in the Supporting materials and methods. In this section, we illustrated the difference when the sample is composed by a retarder and a dichroic element and its inverted ordering. In the case of a sample composed of randomly oriented structures, the polarimetric signature becomes more complex to interpret in the absence of any spatial sectioning. This is shown by the P_d-value reported in Fig. 5 *c*, extracted through LC decomposition, that decreases when the number of fibers increases, from $P_d = 0.97$ with a single fiber to $P_d = 0.54$ with four fibers. Meanwhile, the MM-phasor approach is capable of retrieving the value of R and its orientation without being affected by the degree of P_d. This is only true if we assume that the sample is birefringent, and the influence of depolarization is discussed in the Supporting materials and methods.

In this work, dedicated to the specific case for imaging thin and microscopic birefringent objects, we neglect the presence of any dichroism and depolarization, as reported in Table 1. However, as demonstrated in the case of birefringent randomly oriented multilayers, the sample could exhibit a strong depolarization, making determination of the polarimetric parameters as arduous as it is the for the LC decomposition. We have performed numerical simulations related to the influence on the phasor for a pure dichroic ($D < 0$) and a pure depolarizing ($P_d < 1$) medium ($P_d < 1$ and $R \neq 0°$) presented in the Supporting materials and methods. To sum up, in the case of a pure dichroic medium, the zero-degree orientation angle ($\alpha_D = 0°$) for the linear polarization input phasor presents a modulation value smaller than for a retarder ($M < 0.5$) and becomes maximal on the s axis at $\alpha_D = 45°$. Additionally, the translation in RCP phasor space as a function of α_D is inside the positive g region, as opposed to a pure retarder. Thus, these two pieces of information present an interesting approach for discriminating a pure retarder from a pure dichroic medium. In the case of a pure depolarizing medium, it has been shown that the phasor spot again exhibits a smaller modulation value than for a pure retarder, translating at any orientation to the origin of (g, s) coordinates for highly depolarized samples. This means that for a highly scattering sample ($P_d << 1$) mixed with a retarder, the birefringent parameters cannot be retrieved properly and this phasor approach is limited, as is the case when LC decomposition is applied (36).

Validation on reference sample

We apply the MM-phasor approach on a homemade sample composed of transparent adhesive film surrounded by air, presented in Fig. 6. This sample is of interest because of its strong birefringence signal, its simple model, and the fact that it can be discriminated from air or other weak polarimetric objects. To analyze the phasor plots for the different parts of the sample, we segmented the image separately for the air and the transparent film area by the corresponding ROI 1 (*red*) and ROI 2 (*blue*), respectively, and combined the two objects in ROI 3 (*green*). The arbitrarily chosen ROIs are drawn on the associated m_{00} image of the sample, which corresponds to the total collected light image from the MM.

The phasor for the generated linear and circular polarization states of the air area, ROI 1 (in *red*), is presented Fig. 6 *a*, in accordance with our simulation (Fig. 2). A small difference from the previous simulation comes from the fact that the modulation of the phasor spot is less than 0.5, induced by an R-value between 0 and $\pi/2$. Fig. 6 *b* presents the H and RCP phasors of the transparent film area, ROI 2 (in *blue*), and Fig. 6 *c* corresponds to the area with both air and tape, ROI 3 (in *green*), for comparison. The H phasors for the air and the film regions are almost identical, indicating that the optical fast axis of the film is close to 0°, parallel to the input polarized light. A superposition of the phasors of the two materials is presented in Fig. 6 *c*, corresponding to an ROI taking into account both air and the transparent film area. This is of particular interest because it demonstrates our capacity to provide a distinct segmentation of the different areas in the sample by directly identifying the phasor point distribution in phasor space.

Conjointly, we demonstrate the capability of MM-phasor for converting the phasor (g, s) mapping into the (R, α_R) images. The reconstructed R and α_R images with the histogram distribution of the pixel distribution is presented in Fig. 7, extracted from LC decomposition and from the phasor plots in Fig. 7, *a*and *b*, respectively.

The mean and SD values shown in Fig. 7 *a* correspond to those reported in Table 1 for a homogeneous surface of the tape. The absolute retardance value $R = 2\pi \times \Delta n \times e/\lambda$ of the transparent film is retrieved, knowing its birefringence $\Delta n = 0.0077$ from the coefficients of Cauchy's equation (37) and its approximate thickness $e = 0.5$ mm at the excitation wavelength $\lambda = 808$ nm. Thus, the retardance obtained for such a material corresponds to 60.7 (taking into account the phase wrapping for such a thick birefringent sample), reasonably close to the measured retardance by LC decomposition ($R = 55.5°$). The mean values of R and α_R from the phasor plots are equal to $R = 54.1 \pm 8.4°$ and $\alpha_R = -83.4 \pm 1.5°$, which provide a satisfying agreement with the data from LC decomposition (Fig. 7 *b*). However, we note a stronger dispersion in the values obtained by the phasor plot. In fact, LC is unaffected by changes in transmission, but the phasor approach is affected by undesirable changes in overall transmission. More precisely, LC uses the trace of the retardance Mueller 3 × 3 submatrix (M_r) composed by the block diagonal terms, excluding any attenuation terms (38,39). By comparison, our approach extracts R from the modulation intensity

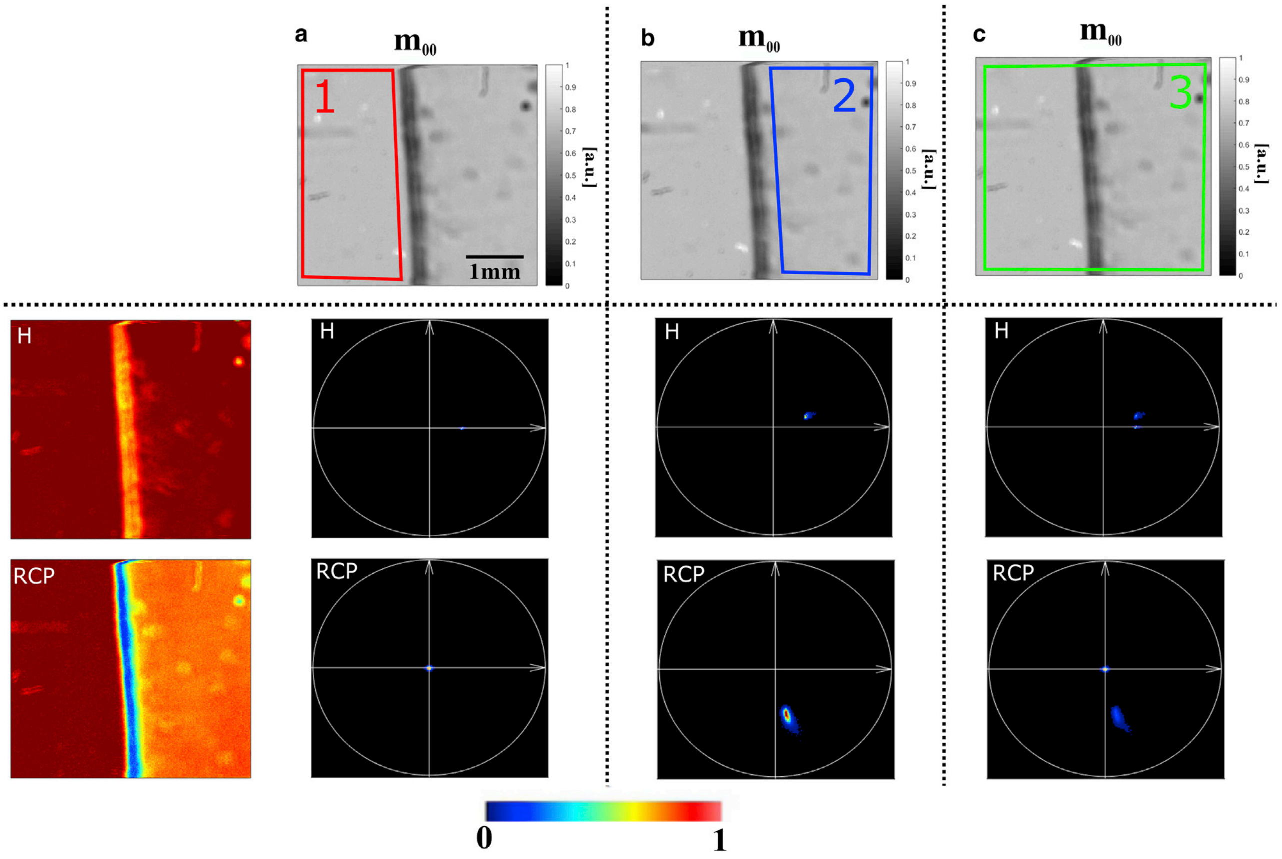

FIGURE 6 Phasor plots of ROIs from a transparent adhesive film sample surrounded by air. (*a*) Phasor plots for the air ROI 1 (*red*), (*b*) phasor plots for the transparent film ROI 2 (*blue*), and (*c*) phasor plots for both air and transparent film tape ROI 3 (*green*) for each of the four input polarization states. (*Top panel*) Images coded in total collected light intensity m_{00}. (*Left panel*) Raw images coded in H and RCP intensity.

performed by a virtual rotation of the analyzer affected by the total transmittance of the sample. This decreases drastically the SNR of such an approach, but this obstacle could be overcome by studying thinner and/or microscopic objects, which is the aim of using the phasor representation presented in the next section.

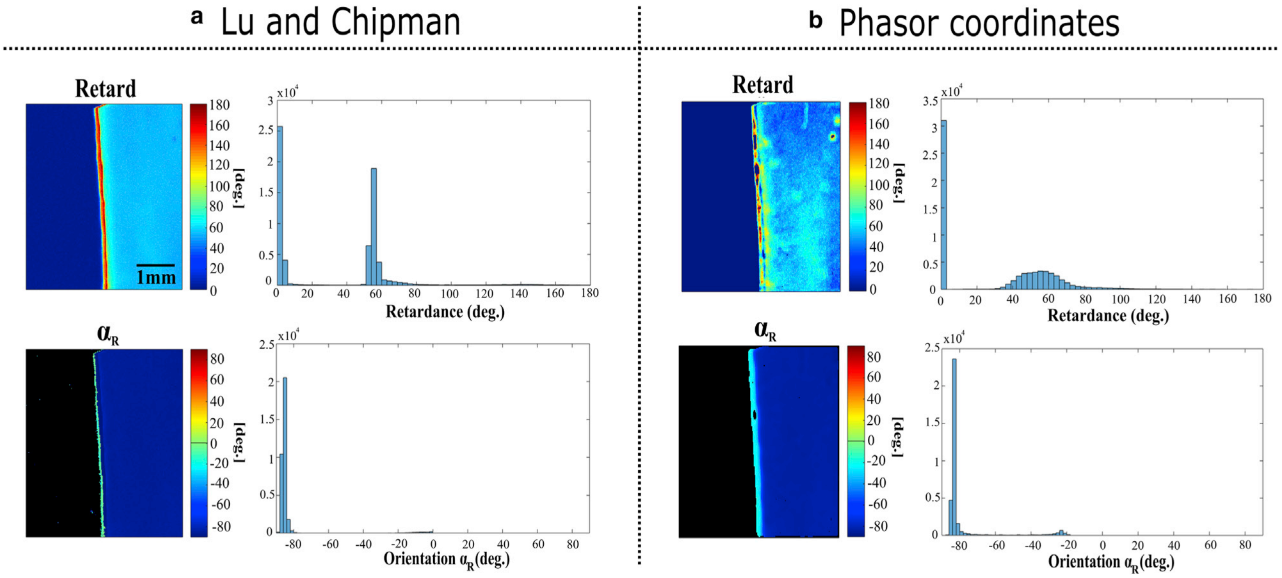

FIGURE 7 (*a*) R and α_R images extracted by LC decomposition and their associated pixel histograms. (*b*) R and α_R images extracted by comparing the H and RCP phasor plots with the associated pixel histogram.

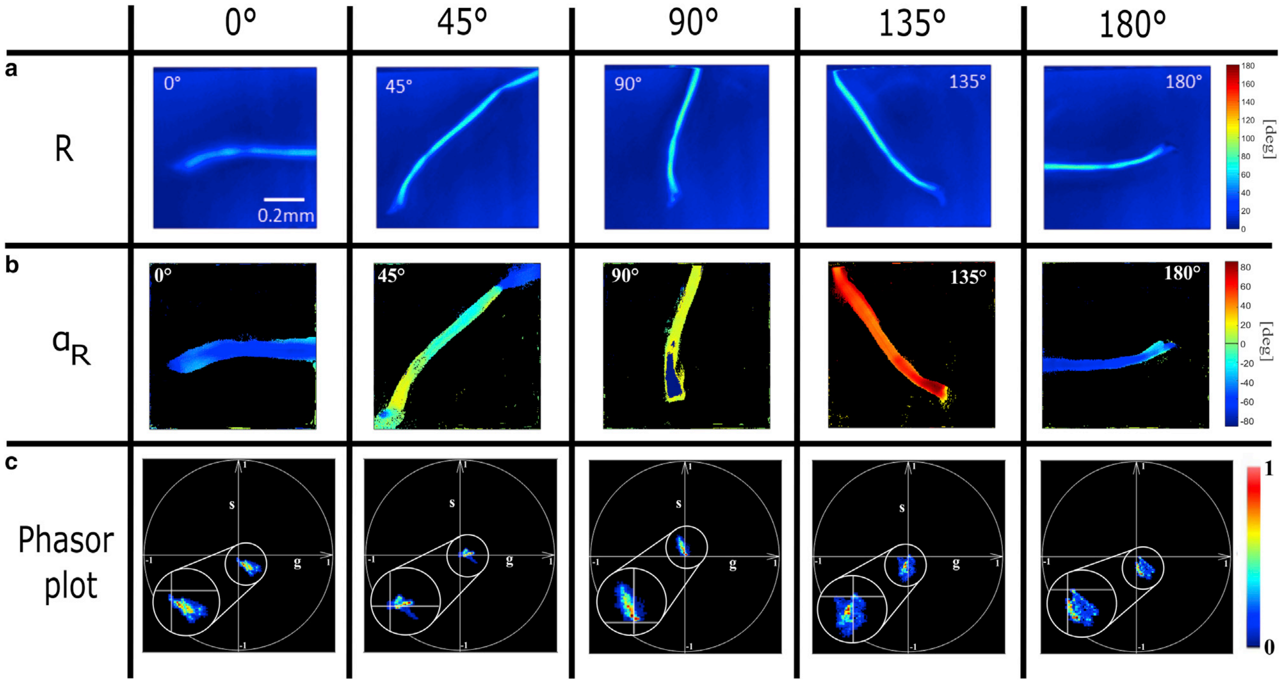

FIGURE 8 (*a*) and (*b*) Retardance images and orientation α_R obtained by LC decomposition of a collagen fiber from rabbit tendon as a function of its spatial rotation ($\in$ [0°, 180°]) at the RCP polarization input. (*c*) Associated RCP phasor plots of the same collagen fiber.

MM-phasor analysis for biological samples

Collagen fiber

As a first application of the MM-phasor, we study a collagen fiber, well-known to be a uniaxial birefringent material (36). Collagen fibers are commonly used as an indicator to diagnose pathologies by detecting tissue disorder using MM imaging (40,41). To understand the influence of the sample orientation relative to the output polarization states, Fig. 8 presents collagen fibers from rabbit tendon after manual physical rotation of the sample. Here, we present the associated LC images, as well as RCP phasor plots for each sample rotation. Because of the weak birefringence effect exhibited by biological samples, the *R*-value is less than $\pi/2$. Therefore, we present only the RCP phasor here.

From the LC images (Fig. 8, *a* and *b*), the *R*-values are similar for different orientations of the fiber, $R = 56.3 \pm 6.9°$, because of the independence of the MM to sample orientation (13). As expected, the associated α_R images in Fig. 8 *b* display shifts of 45° with respect to the physical rotation of the optical fast axis of the sample. The phasor for each of these sample rotations is presented in Fig. 8 *c*. Because of the inhomogeneity and the weaker birefringence of the sample compared with the transparent film, the phasor plot in the practical case exhibits a more scattered spot lying around the center. It is worth noting that the apparent SDs between each rotation of the fiber are explained by an inhomogeneous structure of the sample, coupled with a different z position of the PSF volume between each measurement. However, the spot spreads clearly in a preferential direction resulting from the orientation of the optical fast axis. Additionally, as predicted by Eq. 32, the preservation of the modulation amplitude is explained by the conservation of the *R*-value for all orientations, as shown in LC images (Fig. 8 *a*). We confirm that a sample rotation of $\Delta\alpha_R = 45°$ is

TABLE 2 Averaged mean value of (R, α_R) for collagen fiber as a function of spatial rotation $\in$ [0°, 180°] obtained from LC decomposition and the RCP phasor

Orientation		0°	45°	90°	135°	180°
Parameters						
LC	R	45.4°	58.7°	59.6°	60.9°	64.6°
	α_R	−68.6°	−20.4°	16.3°	55.8°	104.4°
	$\Delta\alpha_R$	N/A	48.2°	36.7°	39.5°	48.6°
RCP phasor	R	69.9°	51.2°	62.3°	56.1°	71.9°
	α_R	−69.2°	−38.2°	9.3°	51.9°	95.6°
	$\Delta\alpha_R$	N/A	31.0°	47.5°	42.6°	43.7°

Collagen fiber presented in Fig. 8, *a* and *b*. RCP phasor shown in Fig. 8 *c*. N/A, not applicable.

characterized by a rotation in the phasor space for the RCP input of $\Delta\phi = 90^\circ$, in keeping with the simulations reported in Fig. 3.

To summarize, Table 2 presents (R, α_R) extracted with the gold standard polarimetric images from LC decomposition and with the RCP phasor plots. These results are obtained by averaging the angle taken from the ROI all along the collagen fiber, taking into account the phase wrapping effect for a physical angle greater than 90°. To confirm the 45° rotation, the differences of α_R between each rotation are presented with $\Delta\alpha_R$.

Table 2 shows an adequate preservation of the R-value for both methods, demonstrating our capability to properly predict the phasor plot behavior with respect to the rotation of the optical fast axis. Indeed, for LC, the averaged R-value for each the sample rotation is $R = 57.9 \pm 7.3^\circ$ and becomes $R = 62.3 \pm 8.8^\circ$ when using the RCP phasor. For the optical fast axis α_R, the averaged difference between each angle, $\Delta\alpha_R = \theta_{i+1} - \theta_i$, is $\Delta\alpha_R = 43.3 \pm 6.0^\circ$ for LC decomposition, whereas it is $\Delta\alpha_R = 41.4 \pm 6.1^\circ$ for the RCP phasor. It is worth noting that for such a sample, the SDs are identical between the two approaches, in contrast to the transparent tape. Indeed, the sample transmittance is much weaker because of its thickness of a few hundred micrometers.

Myosin fiber

As a second application of the MM-phasor, our study of myosin fibers is able to provide an interesting polarimetric signal coming from microscopic-localized change in the complex of actin and myosin (42,43). Fig. 9 presents a myosin tissue sample from rabbit tendon to understand the

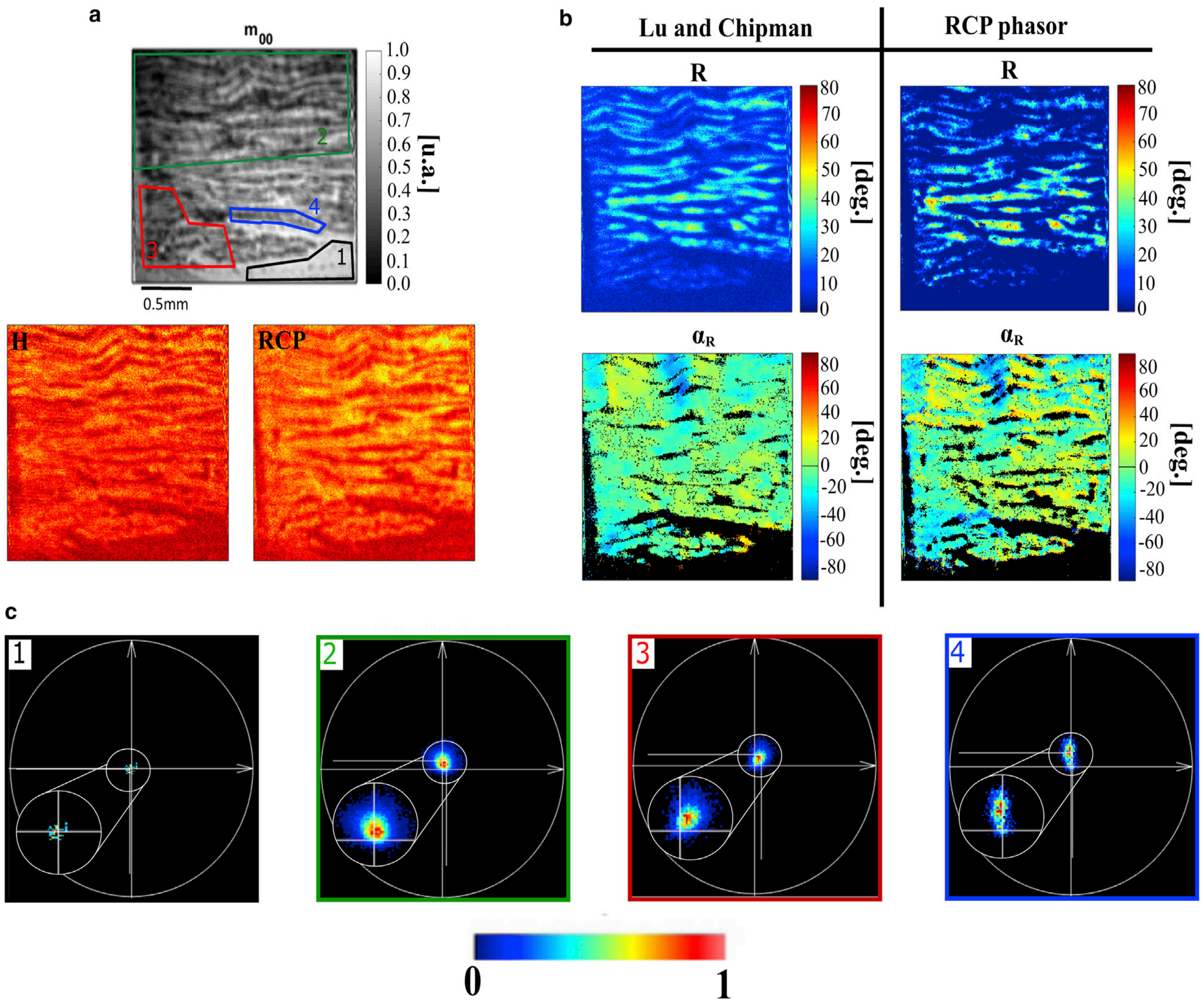

FIGURE 9 (a) Total transmitted light m_{00} image and the raw H and RCP images segmented in four associated ROIs of myosin fibers from a rabbit tendon. (b) The retardance (R) and its orientation (α_R) images from LC decomposition, compared with R and α_R images from the phasor analysis. (c) Phasor plots for RCP input polarization, corresponding to the four ROIs in (a). ROI 1 (*black area*) corresponds to air area, ROI 2 (*green*) to the whole muscular tissue area, ROI 3 (*red*) to the $\alpha_R = -20^\circ$ area of (b), and ROI 4 (*blue*) to the $\alpha_R = 20^\circ$ area of (b).

single influence at the molecular level of the input and output polarization states in the most general case of in situ organized tissue. The reconstructed phasor images, encoded in modulation and phase, are compared with LC decomposition images.

Fig. 9 *a* presents the m_{00} image of the myosin fibers, which provides poor contrast based only on the absorption and scattering of the incoming light. However, after applying LC decomposition, Fig. 9 *b*, left panel presents better image contrast, coded in (R, α_R), in which the fibers can be discriminated according to the orthogonal orientation of the actin-myosin complex. Indeed, it can be observed that the fibers rotate between two orientations, $\alpha_R = -20°$ and $\alpha_R = 20°$, encoded in cyan and yellow, respectively. This symmetrical organization between the actin and the myosin was previously reported in the literature from nonlinear polarization microscopy (44). As expected, images reconstructed from the RCP phasor (Fig. 9 *b*, *right panel*) are in correlation with the structures highlighted by LC decomposition MM images. The RCP phasor plot (Fig. 9 *c*) from ROI 1 (air region), is in accordance with our previous analysis for such reference materials, meaning that no modulation is presented for the RCP input. Nevertheless, for the other ROIs, the phasor spots are slightly wider, probably because of the heterogeneity of the biological components. In particular, in ROI 2, a relatively large region containing fibers of different orientation, the wider spot corresponds to a simple averaging of the overall fingerprint of the muscular tissue. Nevertheless, when segmented separately by ROI 3 and ROI 4, the two oriented fibers reveal clearly two phasors having different phase value at the same modulation (same R-value).

CONCLUSION

In this work, we demonstrate that the combination of a phasor approach with MM microscopy provides an easy and intuitive interpretation of polarization-resolved images from birefringent samples. The method is shown to work when using samples with low dichroic and depolarizing (scattering) content. The impact of using this representation, instead of applying the gold standard LC decomposition, is the absence of a priori knowledge of the sample and of complex mathematical modeling of the elementary polarimetric components. We implemented an algorithm that provides access to the retardance and its orientation in any birefringent material by simply reading the (g, s) coordinates of the linear and circular polarization phasor plots. As preliminary results, we have applied a post-treatment protocol from all the raw polarization-resolved images, resulting in conversion into the phasor map. As future work, the phasor map of each pixel could be accelerated toward a real-time application by directly calculating the (g, s) coordinates for each scanned pixel, which would give an almost immediate result, depending on the scanning time for an image. We show that the MM-phasor can perform fast analysis of tissues, opening possibilities for future applications for in situ diagnosis of pathologies and diseases that could assist histopathological evaluation.

The data sets generated and/or analyzed and the MATLAB algorithm in this work are available from the corresponding author on reasonable request.

SUPPORTING MATERIAL

Supporting material can be found online at https://doi.org/10.1016/j.bpj.2021.06.008.

AUTHOR CONTRIBUTIONS

A.L.G., L.L., and A.D. conceived research, conducted the experiments, and analyzed the results. A.L.G., L.L., A.B., and C.J.R.S. wrote the manuscript. P.B., C.J.R.S., and A.D. contributed to useful discussions. All the authors reviewed and approved the manuscript.

SUPPORTING CITATIONS

Reference (45) can be found in the Supporting material.

REFERENCES

1. Lu, S.-Y., and R. Chipman. 1996. Interpretation of Mueller matrices based on polar decomposition. *J. Opt. Soc. Am. A Opt. Image Sci. Vis.* 13:1106–1113.
2. Bustamante, C., I. Tinoco, Jr., and M. F. Maestre. 1983. Circular differential scattering can be an important part of the circular dichroism of macromolecules. *Proc. Natl. Acad. Sci. USA.* 80:3568–3572.
3. Diaspro, A., M. Bertolotto, …, C. Nicolini. 1991. Polarized light scattering of nucleosomes and polynucleosomes–in situ and in vitro studies. *IEEE Trans. Biomed. Eng.* 38:670–678.
4. Le Gratiet, A., L. Pesce, …, A. Diaspro. 2018. Circular intensity differential scattering (CIDS) scanning microscopy to image chromatin-DNA nuclear organization. *OSA Continuum.* 1:1068–1078.
5. Marongiu, R., A. Le Gratiet, …, A. Diaspro. 2020. ExCIDS: a combined approach coupling expansion microscopy (ExM) and circular intensity differential scattering (CIDS) for chromatin-DNA imaging. *OSA Continuum.* 3:1770–1780.
6. Arteaga, O., and A. Canillas. 2010. Measurement of the optical activity of anisotropic samples by transmission Mueller matrix ellipsometry. *EPJ Web Conf.* 5:03001.
7. Pierangelo, A., A. Nazac, …, A. D. Martino. 2013. Polarimetric imaging of uterine cervix: a case study. *Opt. Express.* 21:14120–14130.
8. Pierangelo, A., S. Manhas, …, P. Validire. 2013. Multispectral Mueller polarimetric imaging detecting residual cancer and cancer regression after neoadjuvant treatment for colorectal carcinomas. *J. Biomed. Opt.* 18:046014.
9. Wang, Y., H. He, …, H. Ma. 2016. Mueller matrix microscope: a quantitative tool to facilitate detections and fibrosis scorings of liver cirrhosis and cancer tissues. *J. Biomed. Opt.* 21:71112.
10. Le Gratiet, A., M. Dubreuil, …, Y. Le Grand. 2016. Scanning Mueller polarimetric microscopy. *Opt. Lett.* 41:4336–4339.
11. Lara, D., and C. Dainty. 2006. Axially resolved complete Mueller matrix confocal microscopy. *Appl. Opt.* 45:1917–1930.
12. Rivet, S., M. Dubreuil, …, Y. Le Grand. 2019. Fast spectrally encoded Mueller optical scanning microscopy. *Sci. Rep.* 9:3972.
13. Le Gratiet, A., M. d'Amora, …, A. Diaspro. 2019. Zebrafish structural development in Mueller-matrix scanning microscopy. *Sci. Rep.* 9:19974.

14. Le Gratiet, A., A. Mohebi, …, A. Diaspro. 2021. Review on complete Mueller matrix optical scanning microscopy imaging. *Appl. Sci. (Basel).* 11:1632.

15. Morio, J., and F. Goudail. 2004. Influence of the order of diattenuator, retarder, and polarizer in polar decomposition of Mueller matrices. *Opt. Lett.* 29:2234–2236.

16. Ossikovski, R., A. De Martino, and S. Guyot. 2007. Forward and reverse product decompositions of depolarizing Mueller matrices. *Opt. Lett.* 32:689–691.

17. Digman, M. A., V. R. Caiolfa, …, E. Gratton. 2008. The phasor approach to fluorescence lifetime imaging analysis. *Biophys. J.* 94:L14–L16.

18. Stringari, C., A. Cinquin, …, E. Gratton. 2011. Phasor approach to fluorescence lifetime microscopy distinguishes different metabolic states of germ cells in a live tissue. *Proc. Natl. Acad. Sci. USA.* 108:13582–13587.

19. Pelicci, S., A. Diaspro, and L. Lanzanò. 2019. Chromatin nanoscale compaction in live cells visualized by acceptor-to-donor ratio corrected Förster resonance energy transfer between DNA dyes. *J. Biophotonics.* 12:e201900164.

20. Ranjit, S., L. Malacrida, …, E. Gratton. 2018. Fit-free analysis of fluorescence lifetime imaging data using the phasor approach. *Nat. Protoc.* 13:1979–2004.

21. Ranjit, S., L. Lanzanò, and E. Gratton. 2014. Mapping diffusion in a living cell via the phasor approach. *Biophys. J.* 107:2775–2785.

22. Fereidouni, F., A. N. Bader, and H. C. Gerritsen. 2012. Spectral phasor analysis allows rapid and reliable unmixing of fluorescence microscopy spectral images. *Opt. Express.* 20:12729–12741.

23. Scipioni, L., E. Gratton, …, L. Lanzanò. 2016. Phasor analysis of local ICS detects heterogeneity in size and number of intracellular vesicles. *Biophys. J.* 111:619–629.

24. Scipioni, L., M. Di Bona, …, L. Lanzanò. 2018. Local raster image correlation spectroscopy generates high-resolution intracellular diffusion maps. *Commun. Biol.* 1:10.

25. Lanzanò, L., I. Coto Hernández, …, G. Vicidomini. 2015. Encoding and decoding spatio-temporal information for super-resolution microscopy. *Nat. Commun.* 6:6701.

26. Sarmento, M. J., M. Oneto, …, L. Lanzanò. 2018. Exploiting the tunability of stimulated emission depletion microscopy for super-resolution imaging of nuclear structures. *Nat. Commun.* 9:3415.

27. Radaelli, F., L. D'Alfonso, …, L. Sironi. 2017. μMAPPS: a novel phasor approach to second harmonic analysis for in vitro-in vivo investigation of collagen microstructure. *Sci. Rep.* 7:17468.

28. Mohebi, A., A. Le Gratiet, …, A. Diaspro. 2021. Combined approach using circular intensity differential scattering microscopy under phasor map data analysis. *Appl. Opt.* 60:1558–1565.

29. Compain, E., S. Poirier, and B. Drevillon. 1999. General and self-consistent method for the calibration of polarization modulators, polarimeters, and Mueller-matrix ellipsometers. *Appl. Opt.* 38:3490–3502.

30. Gil, J., and E. Bernabeu. 1986. Depolarization and polarization indices of an optical system. *Opt. Acta (Lond.).* 33:185–189.

31. Chipman, R. 1994. Hand Book of Optics (Polarimetry), Third Edition. OSA/McGraw-Hill, New York.

32. Stokes, G. 1992. On the composition and resolution of streams of polarized light from different sources. *Trans. Cambridge Philos. Soc.* 9:339–416.

33. Dubreuil, M., F. Tissier, …, Y. Le Grand. 2018. Polarization-resolved second harmonic microscopy of skeletal muscle in sepsis. *Biomed. Opt. Express.* 9:6350–6358.

34. Cloude, S. 1986. Group theory and polarization algebra. *Optik (Stuttg.).* 75:26–36.

35. Silva, D. F., A. S. Gomes, …, M. S. Ribeiro. 2013. Birefringence and second harmonic generation on tendon collagen following red linearly polarized laser irradiation. *Ann. Biomed. Eng.* 41:752–762.

36. Tuchin, V. V. 2016. Polarized light interaction with tissues. *J. Biomed. Opt.* 21:71114.

37. Kinyua, D., G. Rurimo, …, C. Ominde. 2013. Interferometry analysis of cellophane birefringence. *Opt. Photonics J.* 3:337–341.

38. Ossikovski, R. 2012. Differential and product Mueller matrix decompositions: a formal comparison. *Opt. Lett.* 37:220–222.

39. Sheppard, C. J. R., A. Le Gratiet, and A. Diaspro. 2018. Factorization of the coherency matrix of polarization optics. *J. Opt. Soc. Am. A Opt. Image Sci. Vis.* 35:586–590.

40. Ellingsen, P. G., M. B. Lilledahl, …, M. Kildemo. 2011. Quantitative characterization of articular cartilage using Mueller matrix imaging and multiphoton microscopy. *J. Biomed. Opt.* 16:116002.

41. Dubreuil, M., P. Babilotte, …, B. Le Jeune. 2012. Mueller matrix polarimetry for improved liver fibrosis diagnosis. *Opt. Lett.* 37:1061–1063.

42. Codina, M., J. Li, …, S. J. Du. 2010. Loss of Smyhc1 or Hsp90α1 function results in different effects on myofibril organization in skeletal muscles of zebrafish embryos. *PLoS One.* 5:e8416.

43. Bueno, J. M., and M. C. W. Campbell. 2002. Confocal scanning laser ophthalmoscopy improvement by use of Mueller-matrix polarimetry. *Opt. Lett.* 27:830–832.

44. Odin, C., T. Guilbert, …, Y. Le Grand. 2008. Collagen and myosin characterization by orientation field second harmonic microscopy. *Opt. Express.* 16:16151–16165.

45. Azzam, R. M. A. 2011. The intertwined history of polarimetry and ellipsometry. *Thin Solid Films.* 519:2584–2588.

Biophysical Journal, Volume *120*

Supplemental information

Phasor approach of Mueller matrix optical scanning microscopy for biological tissue imaging

Aymeric Le Gratiet, Luca Lanzano, Artemi Bendandi, Riccardo Marongiu, Paolo Bianchini, Colin Sheppard, and Alberto Diaspro

Appendix

Aymeric Le Gratiet[1,*], Luca Lanzano[2], Artemi Bendandi[1,3,4], Riccardo Marongiu[1,3], Paolo Bianchini[1], Colin J.R. Sheppard[1,5], Alberto Diaspro[1,3]

7th June 2021

[1] Nanoscopy and NIC@IIT, Istituto Italiano di Tecnologia, Via Enrico Melen 83, 16152 Genova, Italy
[2] Department of Physics and Astronomy "Ettore Majorana", University of Catania, Via S. Sofia, 64, 95123 Catania, Italy
[3] DIFILAB, Department of Physics, University of Genoa, Via Dodecaneso 33, 16146 Genova, Italy
[4] CONCEPT, Istituto Italiano di Tecnologia, Via Enrico Melen 83, 16152 Genova, Italy
[5] School of Chemistry, University of Wollongong, Wollongong NSW 2522, Australia
* Corresponding author: aymeric.legratiet@iit.it

Averaging the three linear polarization state inputs

The three linear polarization state inputs are oriented at 0° (H), 90° (V) and 45° and generate the phasor plots presented Fig.2. In order to simplify the interpretation of the linear and RCP phasors for the general case, we averaged the phasor plots of the linear polarization states related to the H phasor. The relationship between H and V corresponds to a simple phase shift equal to π with the same modulation, resulting in the new (g,s) coordinates for V

$$g = M * cos(\phi^V + \pi), \quad (1)$$

$$s = M * sin(\phi^V + \pi). \quad (2)$$

To shift the phasor from the 45° to the H input, we have to consider a phase shift and a translation through the s axis in function of R, giving the new coordinates for 45° as

$$g = M * cos(\phi^{45} - \pi/2), \quad (3)$$

$$s = M * sin(\phi^{45} - \pi/2) + 0.5 * (1 + cos(R)). \quad (4)$$

Applying these mathematical transformations, the V and 45° present the same phasor with H, allowing the visualization of the average phasor spots.

Expressions of (R, α_R) in function of (M, ϕ)

Tab.S1 resumes the polarization transformation after interaction with the retarder for R = π, R = $\pi/2$ and R = $\pi/4$ for two orientations of the optical fast axis, such as α_R = 0° and α_R = 45°. It can be predicted by the Stokes-Mueller formalism [1], given by the product of the birefringent sample Eq.28 with the input Stokes vector Eq.12 and Eq.15.

Retarder value	Input polarization	H(1 1 0 0)	CR(1 0 0 1)
**R = π**	α_R= 0°	(1 1 0 0)	(1 0 0 -1)
	α_R= 45°	(1 -1 0 0)	(1 0 0 -1)
**R = π/2**	α_R= 0°	(1 1 0 0)	(1 0 1 0)
	α_R= 45°	(1 0 0 1)	(1 -1 0 0)
**R = π/4**	α_R= 0°	(1 1 0 0)	(1 0 1/√2 -1/√2)
	α_R= 45°	(1 1/√2 0 -1/√2)	(1 -1/√2 0 -1/√2)

Table 1: Output Stokes vectors from the simulated retarder of the three retardance cases R = π, $\pi/2$, $\pi/4$ for θ = 0° and 45° at each input polarization states. The blue shapes correspond to the polarization ellipse.

On one hand, by comparing Tab.1 and Fig.3, the (g,s) coordinates are related to the output polarization states after interaction with the sample by

$$(g, s) = (M * cos(\phi), M * sin(\phi)) = 0.5 * (cos(2\chi).cos(2\psi), cos(2\chi).sin(2\psi)), \tag{5}$$

where χ is the ellipticity of the polarization ellipse ($-\pi/4 \leq \chi \leq \pi/4$), obtained from M = 0.5*cos(2χ) and $\phi = 2\psi$. From an ellipsometric point of view [2], these expressions of g and s are functions of the output Stokes coefficients as

$$(g, s) = 0.5 * (S_1, S_2). \tag{6}$$

Thus, the absolute value of S_3 is retrieved by the expression

$$S_3 = \sqrt{1 - S_1^2 - S_2^2} = sin(2\chi). \tag{7}$$

By converting the MM into the phasor, we lost the direction of rotation of the polarization states defined by the sign of S_3. However, it is always possible to image the S_3 coefficient at the same time as the ones from the modulation amplitude and phase. Thus, the expression of ψ and χ are

$$\psi = 0.5 * tan^{-1}\left(\frac{s}{g}\right), \tag{8}$$

$$\chi = 0.5 * tan^{-1}\left(\frac{S_3}{\sqrt{s^2+g^2}}\right). \tag{9}$$

It is worth noting that the drawback of using the (ψ,χ) images instead of the RCP phasor is the need of using S_3.
On the other hand, from the Mueller matrix formalism, the expression of the transformed polarized light after interaction with the sample can be obtained as a Stokes vector (S_0,S_1,S_2,S_3) using the set of Eqs.12-15 and the MM of the retarder Eq.28. Thus, we can express the Stokes coefficients S_1 and S_2 in function of R and α_R for the RCP input

$$S_1 = sin(R).sin(2\alpha_R), \tag{10}$$

$$S_2 = -sin(R).cos(2\alpha_R). \tag{11}$$

In this way, the last set of equations for the RCP input gives a direct link between (g,s) coordinates and (R, α_R) as follows

$$(g,s) = 0.5 * (S_1, S_2) = 0.5 * (sin(R).sin(2\alpha_R), sin(R).cos(2\alpha_R)). \tag{12}$$

By identifying this last equation with the modulation and phase from the (g,s) coordinates, we show that by simply reading the coordinates (g,s) in the RCP phasor plot, we have the following relation:

$$M = 0.5 * sin(R). \tag{13}$$

It is worth noting that for the phase, the relation is

$$cos(\phi) = sin(\alpha_R) = cos(2\alpha_R + \pi/2), \tag{14}$$

thus,

$$\phi = 2\alpha_R + \pi/2. \tag{15}$$

Pure dichoic medium

The phasor plots for a pure dichroic sample are presented Fig.S1 for the linear and RCP polarization inputs.

For $\alpha_D = 0°$, the modulation value for the H input is smaller than pure retarder cases and becomes maximum (g,s) = (0,0.5) for $\alpha_D < 45°$. In parallel for the RCP input, the modulation is maximum for any orientation but translates from s = 0 to g = 0 in the positive phasor coordinates region, contrary to a pure retarder case (from g = 0 to s = 0 in the negative phasor coordinates region). These two patterns could be used for discriminating between pure retarder and dichroic media. Additionally, the relation between ϕ and α_D is simply

$$\phi = 2 \times \alpha_D \tag{16}$$

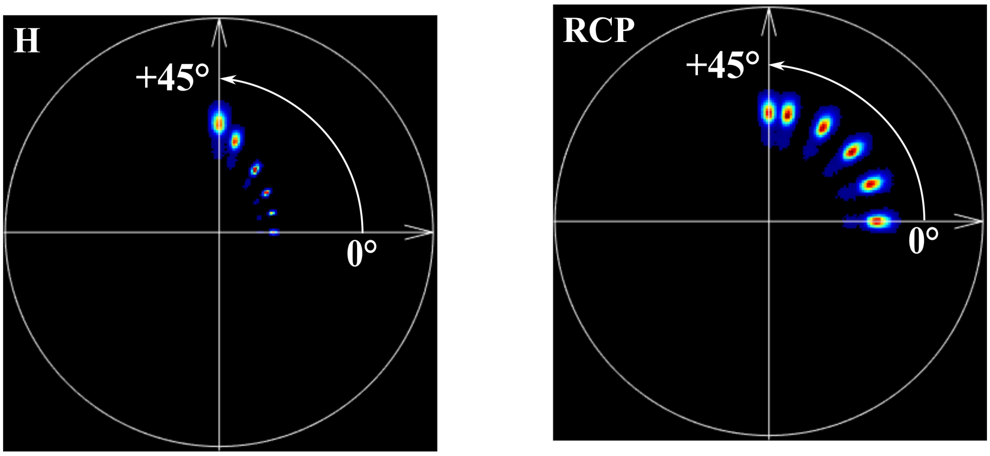

Figure S 1. Phasor plots for a pure dichroic medium at the H and RCP polarization inputs.

Experimental MM	Lu-Chipman
$\begin{bmatrix} 1.000 & -0.027 & 0.006 & 0.003 \\ 0.008 & 0.204 & 0.059 & -0.011 \\ 0.084 & 0.014 & 0.639 & 0.010 \\ -0.002 & 0.058 & 0.017 & 0.065 \end{bmatrix}$	$D = 0.028$<br>$R = 14.85°$<br>$\alpha_R = 44.43°$<br>$P_d = 0.396$
$\begin{bmatrix} 1.000 & -0.043 & 0.002 & 0.001 \\ 0.036 & 0.294 & 0.089 & 0.059 \\ -0.038 & 0.089 & 0.758 & 0.004 \\ -0.003 & 0.021 & -0.024 & 0.039 \end{bmatrix}$	$D = 0.043$<br>$R = 6.34°$<br>$\alpha_R = -38.89°$<br>$P_d = 0.479$

Table 2: Simulated MM of a mixed medium composed by (top) dichroic oriented at 30° and a retarder $\pi/4$ oriented at 30° and (bottom) a retarder $\pi/4$ oriented at 30° and dichroic oriented at 30°.

However, a mixed medium composed by a pure dichroic and a pure retarder leads to a complicated case where the associated polarimetric parameters could be only retrieved by performing several measurements at different sample orientation.

Mixed dichroic and retarder medium

LC decomposition is based on modeling the propagation of the light through a successive arrangement of elementary optical features composed successively by a dichroic, a birefringent and a depolarizer. However, modifying the order with this method leads to misinterpret the real polarimetric signature of the sample and results different value of the parameters. As a demonstration, we evaluate the MM and the associated LC decomposition of a medium composed by a dichroic oriented at 30° followed by a retarder $\pi/4$ oriented at 30°. Then, we extract the MM and the associated polarimetric parameters when this order is inverted as presented Tab.2.

In the meantime, the phasor plot of the two type of media are identical as presented in

Fig.S2.

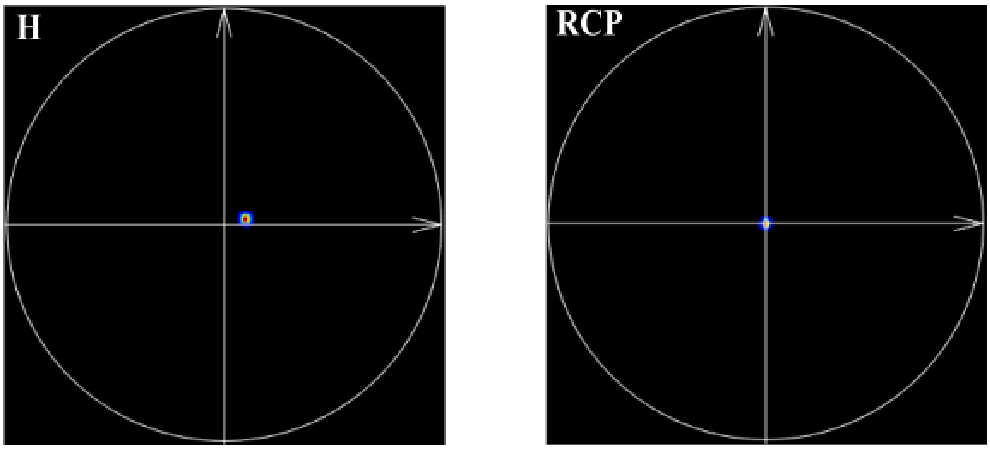

Figure S 2. H and RCP Phasor plots for a mixed medium composed by dichroic oriented at 30° and a retarder $\pi/4$ oriented at 30°.

In summary, we demonstrated that the ordering of the discrete optical features that are used in LC to model the sample is crucial for interpreting the fingerprint of the sample, while there is no influence on the phasor plots since this approach doesn't require any a prio knowledge of the sample.

Pure depolarizing medium

For a depolarizing MM, we calculated the resulting phasor from the R and RCP phasor plot at different value of P_d lying from 1.0 (no depolarizing) down to 0.2 (highly depolarizing) in Fig. 3. We presented the phasor in the case of a mixed polarimetric medium composed by a 90° retarder oriented at 0° and a depolarizer defined by a depolarization index P_d = 1, 0.85, 0.65 and 0.27. As a comparison, the phasor plot in the case of a pure retarder is shown Fig.S3(a) and corresponds to the first phasor spot in Fig.S3(b). The case of a pure depolarizer is presented Fig.S3(c) for P_d = 1, 0.75, 0.5 and 0.2.

Here, we show that the presence of depolarization affects the position of the phasor spot, that results a decreasing of the modulation of the when P_d drops down. In the case of a mixed polarimetric signature birefringent-depolarizer Fig.S3(b), the decreasing of the modulation reduces the contrast between the different phases that could affect the SNR for determining the retardance and its orientation, limiting such approach for highly scattering media. However, it is worth noting that the combined reading of both the H and RCP phasors allows the discrimination overall the possible mixture of retardance and depolarization. For instance, MM-phasor is still capable of distinguishing between a specimen having a small retardance with no depolarization and another one having a high retardance and a strong depolarization. In the extreme case $P_d = 0$ Fig.S3(c), the H and RCP phasor gives both a spot located at the origin, which differs from air sample that is seen at (g,s) = (0.5,0) in the H phasor. A remarkable property of the MM-phasor in dealing with depolarization media is that the (g,s) coordinates for a specific depolarizer is similar of multiplying it by the depolarization factor. Indeed, the Fig.S3(c) gives g = 0.5, 0.38, 0.25 and 0.10 which corresponds to multiplying gmax = 0.5 by Pd = 1.0, 0.75, 0.5 and 0.2, respectively. This simplifies the analysis of the retardance and its

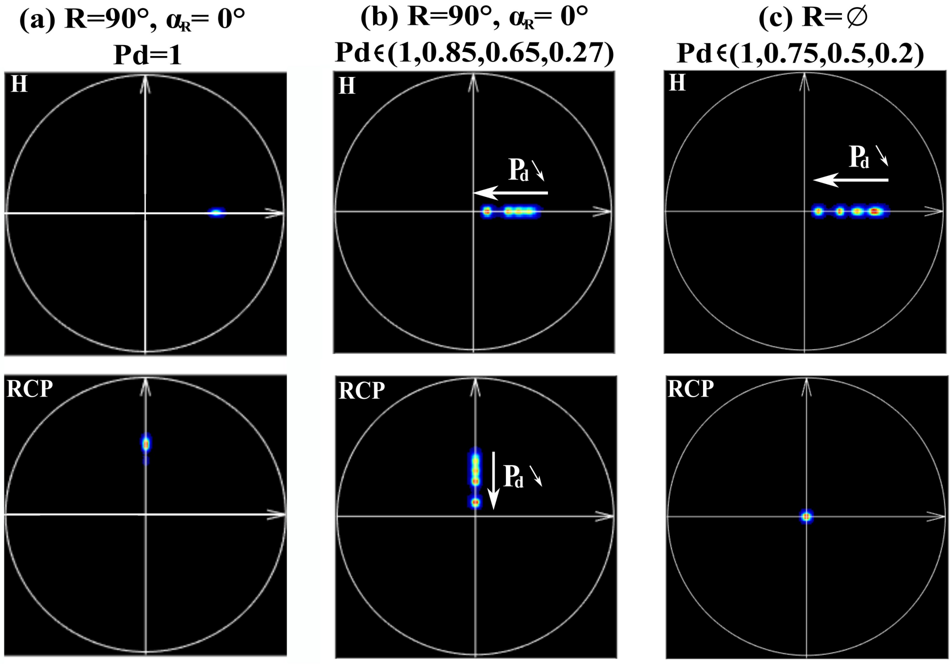

Figure S 3. Phasor plots for (a) A pure retarder (R = 90°, α_R = 90°), (b) a mixed retarder (R = 90°, α_R = 90°) and depolarizer medium and (c) a pure depolarizer. The values for the depolarization index P_d are 1, 0.85,0.65 and 0.27 for (b) and 1, 0.75, 0.5 and 0.2 for (c).

orientation from the phasor plot in the case of depolarization, which is typically the case for biomedical diagnosis of pathological tissue.

References

[1] S. Cloude, "Group theory and polarization algebra," *Optik (Stuttg)*, vol. 75, pp. 26–36, 1986.

[2] R. Azzam, "The intertwined history of polarimetry and ellipsometry," *Thin Solid Films*, vol. 519, no. 9, pp. 2584–2588, 2011.
